# Expression Levels of *hgcAB* Genes and Mercury Availability Jointly Explain Methylmercury Formation in Stratified Brackish Waters

**DOI:** 10.1021/acs.est.2c03784

**Published:** 2022-09-07

**Authors:** Eric Capo, Caiyan Feng, Andrea G. Bravo, Stefan Bertilsson, Anne L. Soerensen, Jarone Pinhassi, Moritz Buck, Camilla Karlsson, Jeffrey Hawkes, Erik Björn

**Affiliations:** † Department of Chemistry, Umeå University, Umeå 901 87, Sweden; ‡ Department of Aquatic Sciences and Assessment, Swedish University of Agricultural Sciences, Uppsala 750 07, Sweden; § Department of Marine Biology and Oceanography, Institute of Marine Sciences, Spanish National Research Council (CSIC), Barcelona 08003, Spain; ∥ Department of Environmental Research and Monitoring, Swedish Museum of Natural History, Stockholm 104 05, Sweden; ⊥ Centre for Ecology and Evolution in Microbial Model Systems—EEMiS, Linnaeus University, Kalmar 391 82, Sweden; # Department of Chemistry, Uppsala University, Uppsala 751 23, Sweden

**Keywords:** mercury, methylmercury, mercury chemical speciation, *hgcAB* genes, *hgcAB* transcripts, metagenomics, metatranscriptomics, Baltic Sea

## Abstract

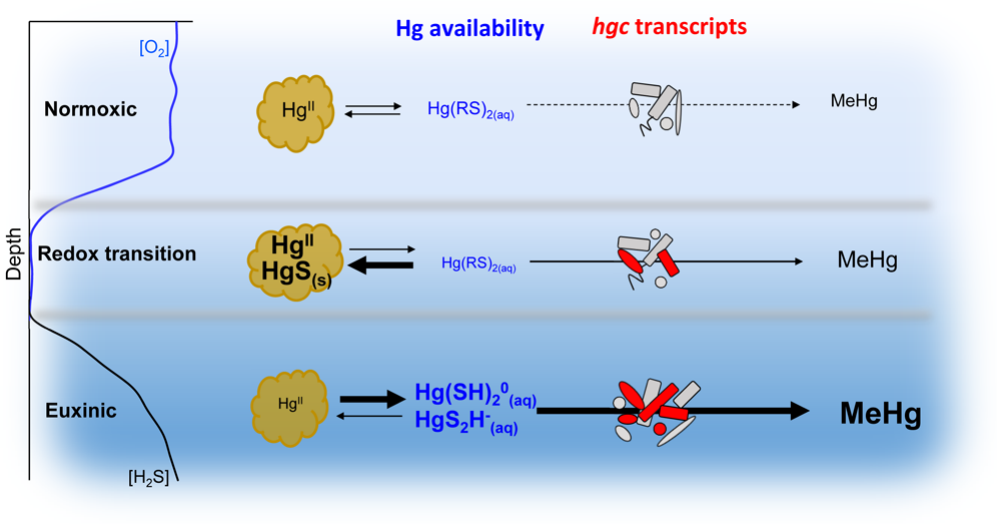

Neurotoxic methylmercury (MeHg) is formed by microbial methylation of inorganic divalent Hg (Hg^II^) and constitutes severe environmental and human health risks. The methylation is enabled by *hgcA* and *hgcB* genes, but it is not known if the associated molecular-level processes are rate-limiting or enable accurate prediction of MeHg formation in nature. In this study, we investigated the relationships between *hgc* genes and MeHg across redox-stratified water columns in the brackish Baltic Sea. We showed, for the first time, that *hgc* transcript abundance and the concentration of dissolved Hg^II^-sulfide species were strong predictors of both the Hg^II^ methylation rate and MeHg concentration, implying their roles as principal joint drivers of MeHg formation in these systems. Additionally, we characterized the metabolic capacities of *hgc*^+^ microorganisms by reconstructing their genomes from metagenomes (i.e., *hgc*^+^ MAGs), which highlighted the versatility of putative Hg^II^ methylators in the water column of the Baltic Sea. In establishing relationships between *hgc* transcripts and the Hg^II^ methylation rate, we advance the fundamental understanding of mechanistic principles governing MeHg formation in nature and enable refined predictions of MeHg levels in coastal seas in response to the accelerating spread of oxygen-deficient zones.

## Abstract 2

This multidisciplinary study reveals the drivers for formation of neurotoxic methylmercury in oxygen-deficient coastal seas. This knowledge is important to predict methylmercury exposure following the warming of the global ocean.

## 1 Introduction

The broad dispersal and local accumulation of mercury (Hg) causes large environmental, socioeconomic, and public health impacts.^1^ Future environmental Hg concentrations and human exposures depend on the success of efforts to reduce anthropogenic Hg emissions by implementing The Minamata Convention on Mercury^2^ and the impact of ecosystem processes on the reactivity and mobility of legacy Hg.^3−5^ The biological formation of methylmercury (MeHg) is a key process as this Hg species is neurotoxic and bioaccumulates in aquatic food webs,^6^ leading to enhanced exposure of Hg to wildlife and humans.^1,7^

In the environment, MeHg is predominantly formed by methylation of inorganic divalent Hg (Hg^II^) and is mediated by microorganisms carrying *hgc* genes (*hgcA* and *hgcB*) coding for a corrinoid protein and a ferredoxin.^8^ Mercury-methylating microorganisms thrive in oxygen-deficient environments (e.g., rice paddies, wetlands, sediments, anoxic waters) in which redox conditions play an important regulating role for both the activity of Hg^II^-methylating microorganisms and the availability of Hg^II^ for methylation.^9^ Beyond sulfate-reducing bacteria,^10^ putative or confirmed Hg^II^ methylators have more recently been reported among iron-reducing bacteria, methanogens, fermenters, and syntrophs,^11−14^ highlighting the large diversity of microorganisms involved in the process.

The expression of genes involved in a specific biological process is a prerequisite for that reaction to happen.^15^ As a consequence, relationships between transcript abundance and the corresponding process have been commonly assumed but rarely observed in the environment.^15^ Although relationships between the abundance of *hgc* genes and MeHg concentrations have been previously identified,^16−19^ no study has yet demonstrated a quantitative relationship between the expression levels of *hgc* genes (i.e., *hgc* transcripts) and Hg^II^ methylation rates in the environment or under laboratory conditions. It is thus uncertain if Hg^II^ methylation rates are constrained by the molecular-level methylation processes mediated by the *hgc* genes. Previous studies have shown that the ability of microorganisms to methylate Hg^II^ can be constitutive^20−22^ and that Hg exposure not necessarily triggers *hgc* gene expression.^20^ Moreover, uncertainties remain about the identity and metabolism of Hg^II^-methylating microorganisms and the specific, and likely variable, environmental drivers that determine their distribution and activity. Accordingly, refined, in-depth sequencing and quantification of *hgc* genes and transcripts as well as other functional genes potentially supporting the Hg^II^ methylation process may provide new information about the distribution and activity of Hg^II^-methylating microorganisms and be important for resolving their roles in Hg^II^ methylation in the environment. As such the reconstruction of metagenome-assembled genomes (MAGs) from environmental samples has been shown to provide meaningful information about the metabolic capacities of putative Hg methylators allowing a better understanding of both environmental factors controlling the presence of *hgc*^+^ microorganisms and potential links between Hg^II^ methylation and other metabolic processes.^13,23−25^

Oxygen deficiency is spreading in coastal seas and the global ocean because of anthropogenic eutrophication and global warming.^26,27^ As one example, the Baltic Sea has seen a dramatic expansion of hypoxic–anoxic water zones over the past 30 years and today holds one of the largest oxygen-deficient sediment areas in the world.^28,29^ The expansion of redox-stratified water columns potentially opens new habitats for Hg^II^ methylators and shifts the availability of Hg^II^ to such microorganisms. Higher MeHg concentrations and molar ratios of MeHg to total Hg (HgT) have generally been observed in oxygen depleted waters of redox-stratified coastal seas,^30−34^ but there are still significant uncertainties regarding factors and processes that control MeHg formation in oxygen depleted coastal waters.

Here, we investigate the relationship between the expression levels of *hgc* genes (i.e., *hgc* transcripts), Hg^II^ chemical speciation (controlling Hg^II^ availability), Hg^II^ methylation rate constants, and MeHg concentrations measured across vertical profiles of the water column at the Landsort Deep and the Gotland Deep in the central Baltic Sea. Both sites have a redox-stratified water column with oxygenated surface water and high dissolved sulfide and MeHg concentrations below the redox transition zone.^34^ We test the hypothesis that the expression of *hgc* genes and the chemical speciation of Hg^II^ control the Hg^II^ methylation rate, and also MeHg concentration, across the redox-stratified water column. We further characterize the metabolic capacities of *hgc*^+^ MAGs to identify major metabolisms associated with putative Hg^II^ methylators in these redox-stratified aquatic systems.

## 2 Materials and Methods

### 2.1 Study Sites

Water samples were collected in late August 2019 in the Baltic Sea at station BY32 (57°19′N 20°03′E) in the Landsort Deep and station BY15 (58°01′N 17°58′E) in the Gotland Deep (Figure S1). Temperature, salinity, oxygen, and sulfide were measured onboard as a part of the Swedish National Monitoring Program. Samples were analyzed according to HELCOM guidelines (HELCOM Combine, 2014, www.helcom.fi) and the quality-controlled data is available in Datasheet 1A and from the website of the Swedish Meteorological and Hydrological Institute (SMHI, 2019, https://sharkweb.smhi.se/hamta-data/). In cases where the determined ancillary parameters did not match our sampling depth, the numerical value of the parameter was determined by linear interpolation from the two nearest depths. Water-sediment interfaces were reached at 204 and 240 m for BY32 and BY15 stations, respectively,

### 2.2 Water Sampling and Sample Handling

Water samples were collected at 6 or 12 sampling depths across the redoxcline using 2 L Niskin bottles (attached to a rosette) at the BY32 and BY15 stations, respectively. Water from the sampling rosette was collected in 2 L fluorinated high-density polyethylene (HDPE) transfer bottles and then split into several bottles for different parameters analysis purposes. All of the sampling vessels were precleaned according to trace metal protocols.^35^ Oxygen-deficient water samples from the redox transition and euxinic zones were pushed out of the Niskin bottles with a N_2_ flow and collected in the transfer bottles inside a N_2_-filled glove bag to avoid exposure to ambient air. HgT samples were collected in 125 mL amber glass bottles (I-CHEM certified 300 series) and MeHg and *k*_meth/demeth_ samples were collected in 250 mL fluorinated HDPE bottles. Samples for HgT and MeHg concentration determination were preserved by addition of 0.1 M HCl and stored in a fridge until analysis.

Incubation experiments to determine the rate constants of Hg^II^ methylation (*k*_meth_) and MeHg demethylation (*k*_demeth_) were conducted onboard following conditions established in Soerensen et al.^35^ Samples were spiked with ^199^Hg^II^- (to 260 pM) and Me^201^Hg (to 2 pM)-enriched isotope tracers and incubated in the dark at room temperature in a N_2_-filled glove box and terminated by acidification to 0.1 M HCl at time points of 0, 4, 8 (or 12), and 24 h. These isotope tracer concentrations are higher than ambient Hg concentrations to ensure detectable MeHg concentrations but are in the lower end of concentration ranges typically used to determine MeHg formation and degradation in natural waters.^34,36,37^

For DNA and RNA analysis, three 1 L water samples were collected totally at six depths from the normoxic (1 sample), redox transition (2 samples), and euxinic water layers (3 samples) at the two stations. Sampling and filtration equipment were sterilized with 5% bleach and rinsed with Milli-Q water. Gosselin HDPE plastic bottles (Fisher Scientific UK Ltd.) were used only once for each sampling. Sampling blanks (SC1, SC2) consisting of 1 L bottles filled up with Milli-Q water and open during sampling at each station were used for molecular analysis. The sampling depths corresponded to that for chemical parameters. All samples were filtered through 0.22 μm sterivex poly(ether sulfone) enclosed filters using a peristaltic pump. After filtration, RNAlater solution was added to each filter and the filters were stored at −20 °C prior to DNA and RNA extractions.

### 2.3 Determination of HgT and MeHg Concentrations and *k*_meth_ and *k*_demeth_

The HgT water samples were treated with BrCl, and Hg^II^ was subsequently reduced to Hg^0^ by SnCl_2_ and stripped off by purging with N_2_ followed by dual gold amalgamation and cold vapor atomic fluorescence spectroscopy (CVAFS, Tekran Mercury Detector 2500) detection (US-EPA Method 1631 E). Lab blanks consisting of MQ water (*N* = 6) were lower than the limit of quantification (LOQ) (0.25 pM). Field blanks (*N* = 6) were prepared from MQ water, brought onboard the cruise and treated as samples, and the determined concentration was 0.36 ± 0.07 pM. We subtracted the difference in the field and lab blanks (0.11 pM) from all HgT samples for blank correction. The relative standard deviation (RSD) of triplicate field samples collected from the same water depths was an average of 12%.

MeHg concentrations and *k*_meth/demeth_ rate constant samples were spiked with a Me^200^Hg isotope-enriched internal standard in the lab, derivatized with sodium tetraethylborate, purged and trapped on a Tenax adsorbent,^38,39^ and subsequently determined by isotope dilution analysis using thermal desorption gas chromatography inductively coupled plasma mass spectrometry (TDGC-ICPMS, Markes-100 TD unit, Agilent 7980 GC system, Agilent 7700 ICPMS system) measurements. The MeHg sample concentrations were blank corrected by subtracting half the limit of detection (LOD) value, which was 49 fM based on 3 times the standard deviation of low MeHg concentration samples (*n* = 11). The RSD of triplicate field samples collected from the same water depths was an average of 4%. The *k*_meth_ and *k*_demeth_ rate constants were determined as the slope of linear fits of the Me^199^Hg concentration or ln(Me^201^Hg/Me^201^Hg_*t*=0_) concentration ratio, respectively, versus time. Rate constants were calculated only for samples with a statistically significant linear regression (*p* < 0.05, Figure S2) and were considered nondetectable for other samples. It should be noted that the exact detection limit values for *k*_meth_ and *k*_demeth_ are sample-specific (dependent, e.g., on ambient Hg species concentrations^40,41^). We estimated a typical detection limit for *k*_meth_ to 0.016 × 10^–3^ h^–1^, calculated from 3 × SD of the Me^199^Hg concentration in the samples at time point 0 h (which theoretically should have a Me^199^Hg concentration of 0 fM). The typical detection limit for *k*_demeth_ was estimated to be 4 × 10^–3^ h^–1^, calculated from a 10% decrease in the average Me^201^Hg/Me^201^Hg_*t*=0_ ratio of the samples at time point 0 h.

### 2.4 Hg Speciation Model

The chemical speciation of Hg^II^ and MeHg across the different water zones was determined by thermodynamic modeling using WinSGW software from Majo, Umeå, Sweden.^42^ We adapted a model developed by Liem-Nguyen et al.,^43^ which comprised 7 components and 24 species. Specifically, we used Hg species and stability constants from the model by Liem-Nguyen et al.^43^ but adjusted the pH, ionic strength, and concentration of all components to fit the Baltic Sea environment. A full description of the model used in our study is given in Tables S1 and S2. The inputs to the model were the determined concentrations of Hg^II^ (calculated as HgT–MeHg), MeHg, H_2_S, and fixed Cl^–^ (90 mM) and pH (7.3) values, as well as the estimated concentration of thiol functional groups associated with dissolved organic matter (DOM), as 0.15% of the dissolved organic carbon (DOC) concentration.

### 2.5 DOM Composition

Dissolved organic matter (OM) was extracted from water samples according to the method by Dittmar et al.^44^ The sample volumes varied from 350 to 1000 mL, and DOM extracts were prepared by solid-phase extraction so that approximately the same equivalent volume of seawater was analyzed in each case. The methanol extracts were dried down, redissolved in 5% methanol in water, and analyzed by liquid chromatography–mass spectrometry (LCMS) using a Polar C18 column (Phenomenex) with detection using a diode array detector (DAD) (light absorption), charged aerosol detector (material abundance), and negative mode electrospray ionization mass spectrometry (Orbitrap^45^). To evaluate the DOM composition from the water samples, the intensities throughout the chromatographic separation were summed to produce one peak list for each sample (Figure S3), to which formulas were assigned (Figure S4). Additionally, Suwannee River Fulvic Acid (International Humic Substances Society batch 2S101F) was analyzed for quality control before and after the samples. In total, 1840 chemical formulas were found in at least one sample in the dataset. The data were normalized in each sample to a sum of 1 × 10^6^, and samples were compared pairwise using the Bray–Curtis dissimilarity metric (Figure S5). The dissimilarity matrix was used to perform a principal coordinate analysis (Figure S6).

### 2.6 Molecular Analysis and Bioinformatics

DNA and RNA were extracted from one and two filters, respectively, with the FastDNA SPIN Kit for the soil filter and two with the QIAGEN RNeasy mini kit. Blanks were used during DNA extractions (exB1, exB2). The TURBO DNA-free kit (Invitrogen) was used to DNase treat extracted RNA and remove DNA contamination and afterward controlled for residual DNA by a 30-cycle polymerase chain reaction (PCR) with 16S rDNA primers (27F and 1492R) including Milli-Q as negative and DNA from *Escherichia coli* as positive controls. This was followed by bacterial ribosomal RNA depletion using a RiboMinus Transcriptome Isolation Kit (Thermo Fisher Scientific). The final quantity and quality of extracted nucleic acids were measured on a NanoDrop One spectrophotometer (Thermo Fisher Scientific) and a qubit. Library preparation of DNA and RNA for sequencing was prepared with the ThruPLEX DNA-seq (Rubicon Genomics) and TruSeq RNA Library Prep v2 (Illumina) kits, respectively. The DNA and RNA samples were each sequenced with a paired-end 2 × 150 bp setup at the Science for Life Laboratory (Stockholm) at the SNP&SEQ Technology Platform over an entire Illumina NovaSeq6000 SP4 flow cell. The raw data are accessible at NCBI (SRA accession: PRJNA627699). The bioinformatic procedure is described in detail in the Supporting Information.

### 2.7 Statistics

Bivariate correlations were calculated between environmental and biological parameters as the Spearman’s rank using a 2-tailed test of significance using the function *rcorr* from the R package Hmisc (v3.6.1^46^) and visualized using the function *corrplot* from the R package corrplot (v0.90^47^).

## 3 Results and Discussion

### 3.1 Contrasting Redox Transition Zones at the Landsort Deep and the Gotland Deep

**Figure 1:**
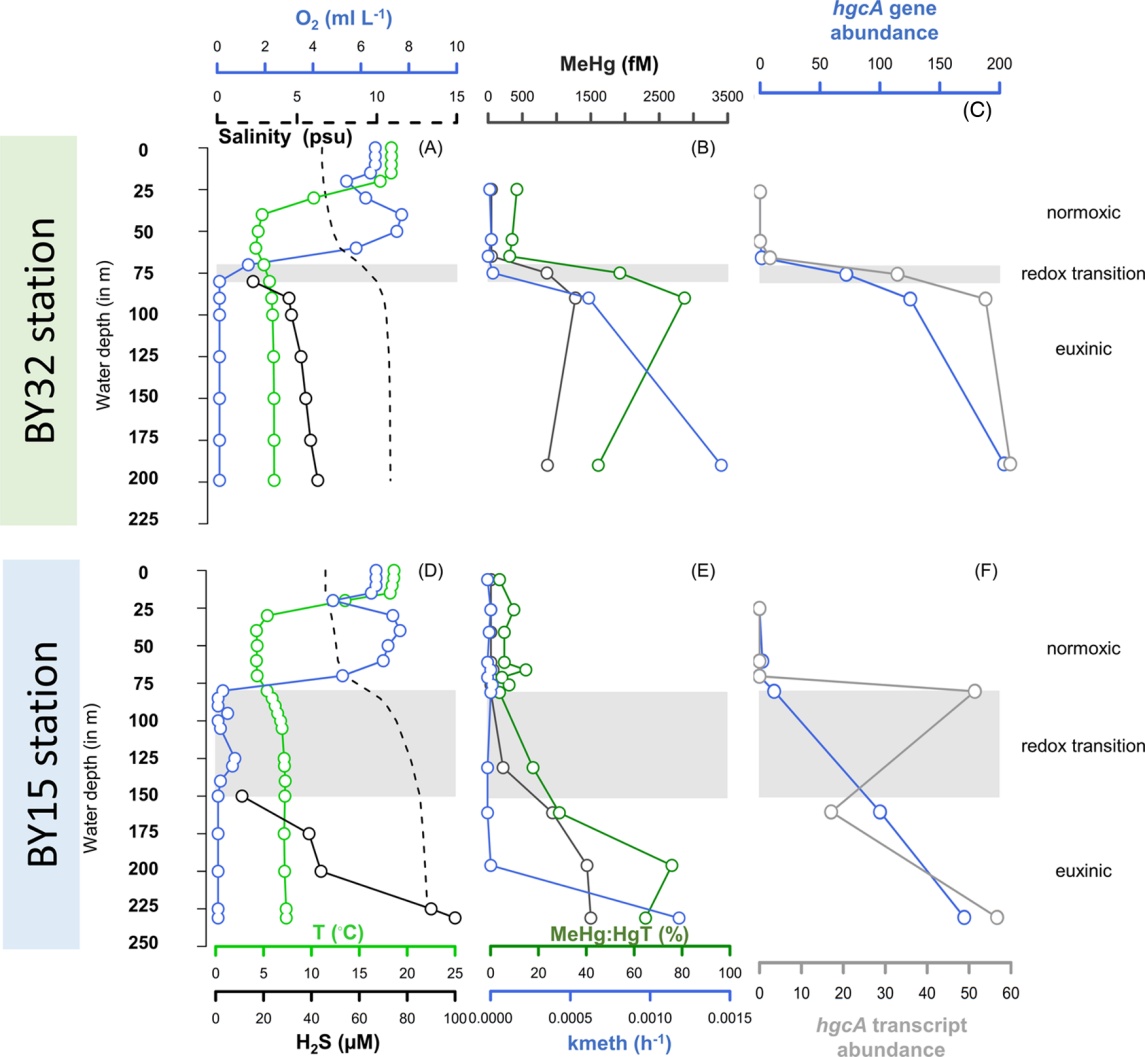
Water depth profiles of ancillary parameters (temperature, salinity, oxygen, hydrogen sulfide) (A, D); MeHg concentrations, MeHg/HgT molar ratio (%), and Hg^II^ methylation rate constant (*k*_meth_) in unfiltered water samples (B, E); and *hgcA* gene and transcript abundance (coverage values in reads/bp normalized with mean coverage values from the housekeeping gene *gyrB*) (D, F) for stations BY32 (A–C) and BY15 (D–F).

In the central Baltic Sea, pelagic redoxclines are present in several locations, including the Landsort Deep and Gotland Deep where we collected water samples in August 2019 at stations BY32 and BY15, respectively (Figure S1). Thermoclines were present at 15–40 m water depths for both stations and haloclines at 50–90 and 60–150 m at BY32 and BY15 stations, respectively (Figure 1A,D and Datasheet 1A). Normoxic water layers (defined as O_2_ concentrations >2 mL L^–1^) were present from the surface to ∼60 and ∼70 m depths at the BY32 and BY15 stations, respectively. At BY32, the redox transition zone (here defined as water with O_2_ concentrations between 0.1 and 2 mL L^–1^) was sharp and distinct from ∼70 to 80 m. In contrast, the redox transition zone was more extended and distorted reaching from ∼80 to 150 m at BY15. The reason for this difference between the two stations is turbulent mixing with oxygenated saline water inputs from the North Sea moving below the halocline to the Gotland basin. These processes substantially impact the redoxcline^34,48^ in the Gotland Deep (BY15 station) but less so in the Landsort Deep (BY32 station; Figure S1). Correspondingly, euxinic conditions (defined as water with <0.1 mL L^–1^ O_2_ and detectable H_2_S) appeared already at ≤ 80 m at BY32 but at ∼150 m at BY15. At greater depths, H_2_S concentrations exceeded 30 μM at both stations and reached a maximum of 100 μM below 200 m at the BY15 station.

### 3.2 Hg^II^ Methylation Rate Constants and MeHg Concentrations in Relation to Redox Conditions

The Hg^II^ methylation rate constant (*k*_meth_), determined in incubation assays with an isotopically enriched ^199^Hg^II^ tracer, was not detectable (<0.030 × 10^–3^ h^–1^) in normoxic water samples but increased throughout the euxinic zone and followed the H_2_S concentration reasonably well (Figure 1). At BY32, the *k*_meth_ value was close to the detection limit (0.016 × 10^–3^ h^–1^) in the lower part of the redox transition zone (75 m depth) and then increased substantially with depth and H_2_S concentration in the euxinic zone (0.63 × 10^–3^ and 1.47 × 10^–3^ h^–1^ at 90 and 190 m, respectively). At BY15, the *k*_meth_ value was below the detection limit throughout the water column until the largest depth in the euxinic zone where a high *k*_meth_ value was observed (1.20 × 10^–3^ h^–1^ at 230 m). MeHg demethylation rate constants (*k*_demeth_) for samples incubated in the dark were detectable (detection limit 4 × 10^–3^ h^–1^) only for the greatest depth at each station (7.6 × 10^–3^ h^–1^ at BY32, 190 m and 5.1 × 10^–3^ h^–1^ at BY15, 230 m, Datasheet 1A).

The vertical distributions of HgT and MeHg concentrations and the molar ratio of MeHg/HgT measured in the water samples followed the same overall vertical distribution across the three redox zones as *k*_meth_ and increased in the order: normoxic < redox transition < euxinic water zones (Figure 1B,E and Datasheet 1A). While the HgT concentration increased by a factor of ∼4 from normoxic to euxinic waters (from 290–640 to 1800–3000 fM), the increase in the MeHg concentration was considerably higher with a factor of ∼23 (from <50 to 1000–1300 fM). The MeHg/HgT molar ratio increased accordingly and was <17, 21–60, and 50–90% in the normoxic, redox transition, and euxinic water zones, respectively. Overall, the HgT and MeHg concentrations and MeHg/HgT molar ratio observed in our study were in the same ranges as previous observations for redox-stratified waters in the Baltic Sea,^31,34^ although Soerensen et al.^34^ observed maximum HgT concentrations that exceeded the levels measured in our study. Differences in the depth of the maximum MeHg concentration and/or MeHg/HgT ratio have been reported among or within studies on redox-stratified coastal systems, with maxima located either at the interface between the redox transition (or hypoxic) zone and the euxinic (or anoxic) zone or in deeper euxinic water layers with H_2_S concentrations in the μM range.^30−34,49^ Notably, the MeHg concentration and MeHg/HgT ratio, which are the parameters normally determined in studies on MeHg in coastal seas, are proxies for the net formation of MeHg.^50^ These parameters thus represent the net result of Hg^II^ methylation and MeHg demethylation (and are in some cases also influenced by the input and output of Hg^II^ and MeHg in the studied system) and do not enable us to study the two processes separately. There are very few studies that determine the individual rate constants *k*_meth_ and *k*_demeth_ in redox-stratified coastal seas.^34^

Our study demonstrates that MeHg gross formation (i.e., methylation rate of Hg^II^) is the largest in euxinic waters with a high H_2_S concentration, rather than at the interface between the redox transition and euxinic zones. Our results further suggest that the MeHg concentration and MeHg/HgT ratio in the oxygen-deficient water zones are mainly controlled by in situ Hg^II^ methylation, possibly with an exception at the deepest euxinic water where also MeHg demethylation was high. These results highlight the value of the rate constants determined for Hg^II^ methylation and MeHg demethylation along with the MeHg concentration and MeHg/HgT ratio to fully characterize MeHg cycling in coastal seas.

### 3.3 Chemical Speciation Modeling Revealed the Highest Availability of Hg^II^ for Methylation in the Euxinic Zone

The chemical speciation of Hg^II^ in the different pelagic redox zones in the Central Baltic Sea have previously been described conceptually and by thermodynamic modeling.^34^ We used the same thermodynamic model to verify the Hg^II^ speciation for the conditions at the time of our sampling cruises (Table S1) but improved the accuracy by including DOC observations from the cruise rather than the generic values used in Soerensen et al.^34^ The results of the model (Table S2) suggest that in normoxic waters, Hg^II^ is present as complexes with thiol groups associated with dissolved or particulate OM. In the redox transition zone, the model predicts that Hg^II^ complexes with thiols dominate at H_2_S concentrations lower than ∼0.003 μM. These Hg^II^–thiol complexes are expected to predominantly be associated with particulate aggregates consisting of Fe^III^-oxyhydroxides and OM in the transition zone.^30,34,51^ The solid-phase metacinnabar, HgS(s), dominates the Hg^II^ speciation at H_2_S concentrations in the fairly narrow range (in absolute terms) of 0.003–0.25 μM, whereas dissolved Hg^II^-sulfide species outcompete HgS(s) at H_2_S concentrations exceeding 0.25 μM. Notably, thermodynamic constants are not available for nanoparticulate HgS clusters but such species would only be stable in an even more narrow concentration range of H_2_S given the higher solubility of such phases compared to crystalline metacinnabar.^52^ The distribution among dissolved Hg^II^-sulfide species are 86.5% HgS_2_H^–^, 10.1% Hg(SH)_2_, and 3.4% HgS_2_^2–^ at pH 7.3, as used in our model, irrespective of the H_2_S concentration above 0.25 μM.

It is important to note that the particulate Hg^II^ phases present in the redox transition zone have low availability for microbial Hg^II^ methylation compared to the dissolved Hg^II^-sulfide species dominating in the euxinic water zone.^53,54^ Previous studies proposing maximum MeHg formation at the interface of redox zones based on the MeHg concentration or MeHg/HgT ratio (i.e., proxies for MeHg net formation) have explained this observation with a decreased Hg^II^ availability for methylation at high μM concentrations of H_2_S. These conclusions, however, were partly based on inaccurate speciation modeling of Hg^II^ with models largely influenced by the species HgS^0^(aq),^55^ which has been refuted in later studies,^56,57^ and/or based on the speciation modeling of soils and sediment with orders of magnitude higher Hg^II^ concentrations than in marine waters. In contrast, our speciation modeling predicted the highest availability for methylation of Hg^II^ in the euxinic zone, at H_2_S concentrations exceeding 0.25 μM, through the formation of dissolved Hg–sulfide species. This result is in line with the highest observed *k*_meth_, MeHg concentration, and MeHg/HgT ratio in the euxinic zone in our work.

### 3.4 Abundance and Activity of Hg^II^-Methylating Taxa Is Tightly Linked to Redox Conditions

**Figure 2:**
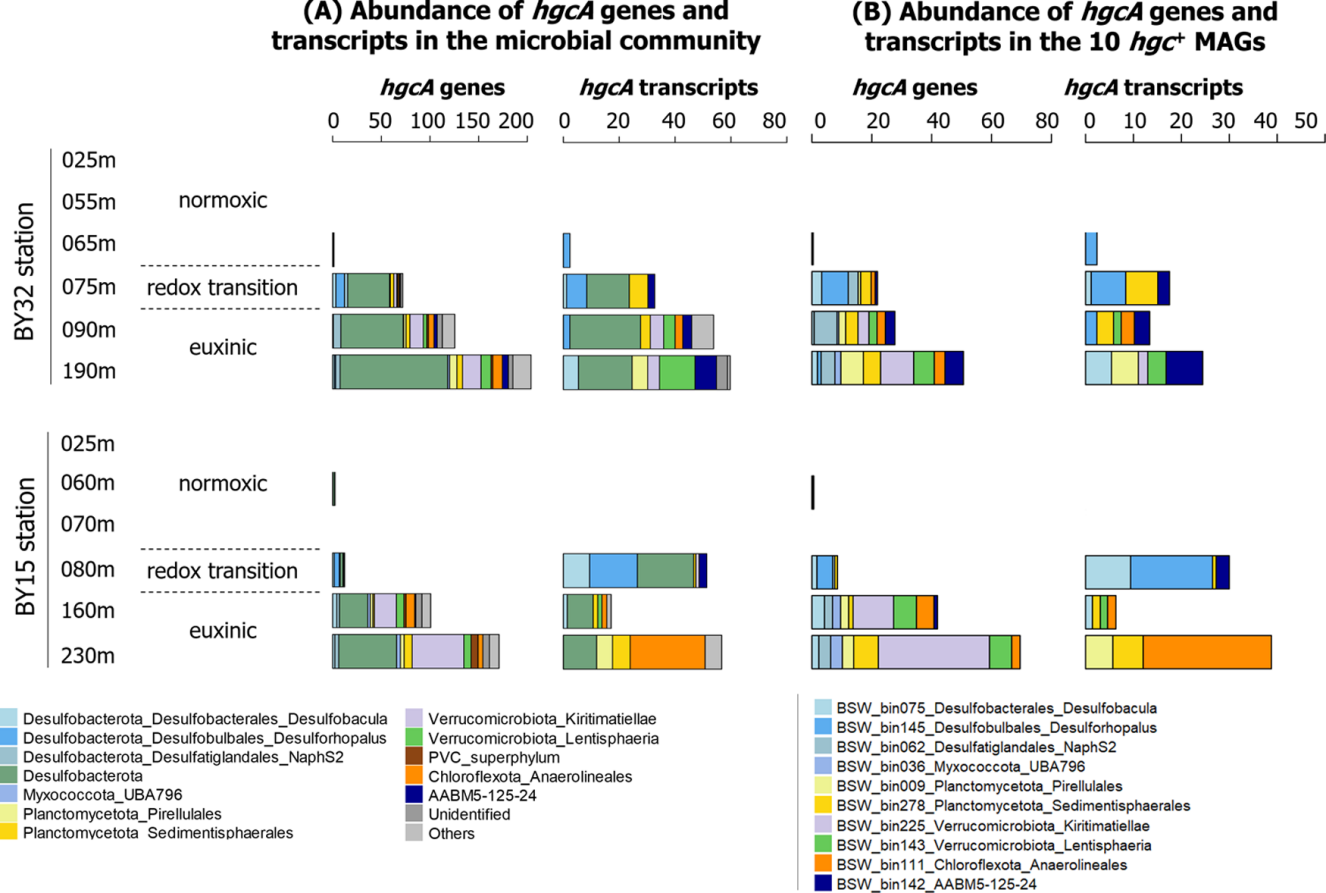
Distribution of *hgcA* genes in terms of abundance (coverage values in read/bp normalized with mean coverage values from the housekeeping gene *gyrB*) of both total genes and transcripts for all *hgcA* genes detected in the water metagenomes (A) and the 10 *hgcA*^+^ MAGs (B). Color codes correspond to the taxonomic identification of each microbial group, see bottom panels for correspondence.

Previous metagenomic studies have demonstrated the presence of *hgc* genes in oxygenated marine pelagic systems, suggesting a potentially important role of certain aerobic lineages, e.g., *Nitrospina*, in marine Hg^II^ methylation.^58−60^ So far, less attention has been given to oxygen-deficient marine environments^25,61,62^ where other Hg^II^-methylating lineages are expected to thrive. Consistent with other recent findings,^61^ our results based on the sequencing of metagenomes and metatranscriptomes (Datasheet 1B) from water samples demonstrated that redox conditions are a major factor controlling the presence and abundance of potential Hg^II^ methylators in the central Baltic Sea water column (Figures 1 and 2). An overview of the link between redox conditions and the composition and metabolism of the overall microbial community is described in the Supporting Information (including Figure S7).

Using metagenomes and metatranscriptomes, we focused here on the study of the distribution and activity of microorganisms carrying and expressing *hgc* genes, and thus, potentially involved in Hg^II^ methylation (Figure S8). In terms of abundance (i.e., normalized coverage values as described in Section 2), the overall pattern was similar at both stations. The *hgcA* (Figure 2A and Datasheet 1C) and *hgcB* (Figure S9 and Datasheet 1C) genes and their transcripts were either absent or at very low levels in the normoxic zone, increased in the redox transition zone, and were present in the highest abundances in the euxinic zone. One exception to this general pattern was the lower abundance of *hgc* transcripts at BY15 160 m as compared to other samples from the euxinic zone (Figures 2A and S9). Analogous to the redox and Hg parameters, the increase in the *hgc* gene and transcript abundances across the redox zones were sharper at the BY32 compared to the BY15 station. At both stations, *hgc* transcripts from members of Desulfobacterota and PVC superphylum (i.e., Planctomycetota, Verrucomicrobiota) dominated in both the redox transition and euxinic zones, while *hgc* transcripts from Chloroflexota and AABM5-125-24 were only abundant in euxinic waters samples (Figures 2A and S9).

**Figure 3:**
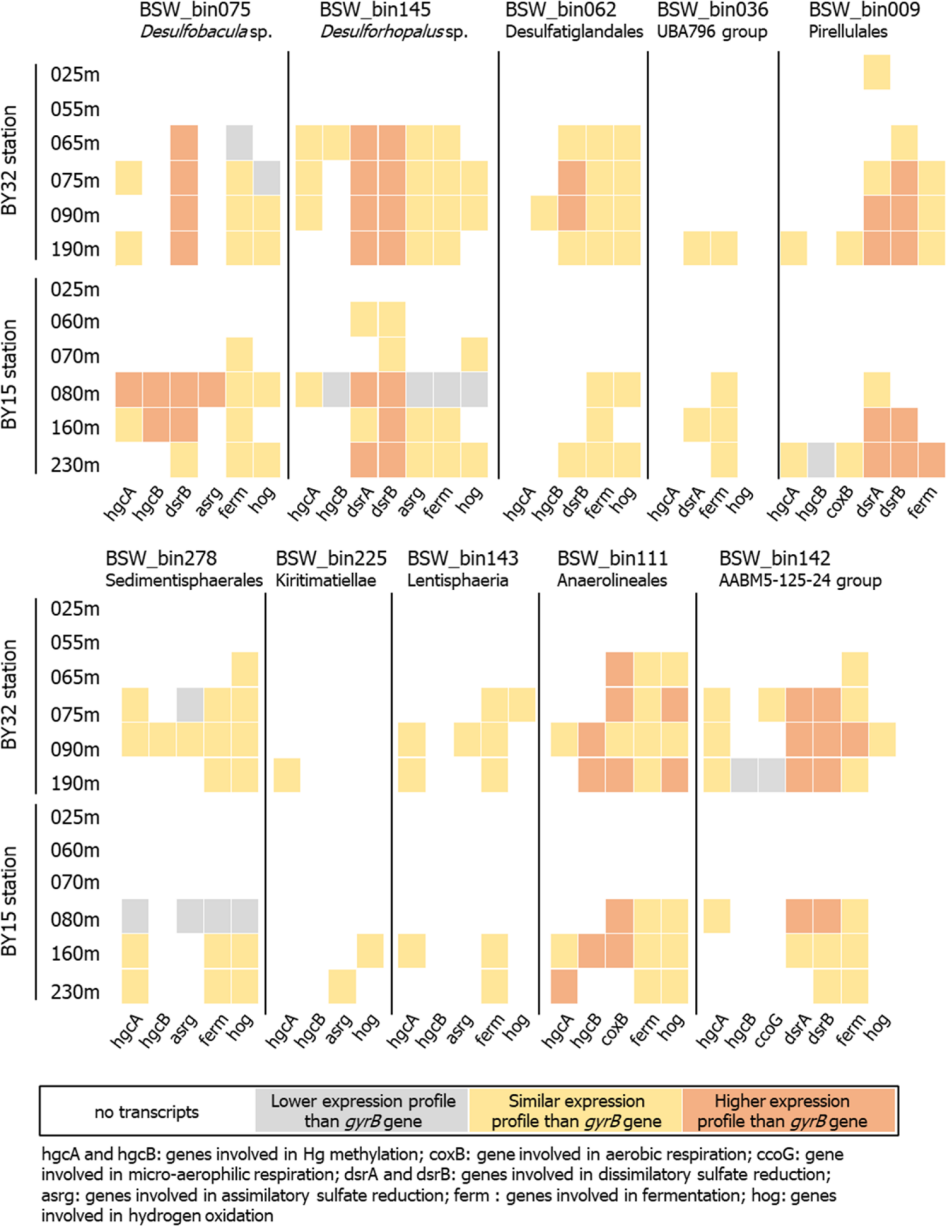
Expression profiles of functional genes by *hgc*^+^ MAGs found in BY32 and BY15 stations. For each MAG, expression levels were calculated comparing the coverage values of transcripts from functional genes to the coverage values of the transcripts from the housekeeping gene *gyrB*. Colors denote expression levels of functional genes lower (gray), similar (yellow), and higher (orange) than the expression levels of *gyrB* genes. Expression levels were considered similar if the differences between coverage values were less than 0.005 (see Datasheet 1G for exact values).

The reconstruction of 127 metagenome-assembled-genomes (MAGs; Datasheet S1E) revealed that 10 of them carried *hgc* genes that contributed to, respectively, 44 and 59% of the *hgcA* and *hgcB* transcripts found in the euxinic zone from both stations (Datasheet 1C). A detailed characterization of these 10 putative Hg^II^ methylators and their metabolic traits can inform about the metabolic versatility of microorganisms that are capable of Hg^II^ methylation and about environmental factors that control their distribution and activity.^9^ To investigate the expression levels of *hgc* genes found in MAGs, we compared the expression level of *hgc* and metabolic capacity genes to expression levels of the housekeeping gene *gyrB* detected in the 10 *hgc*^+^ MAGs (Figure 3). For each MAG, expression levels were considered as high if they surpassed the *gyrB* expression level by 0.005 (in coverage values of transcripts).

We found that *hgc* genes were expressed in the redox transition and euxinic zones by MAGs featuring in total five different metabolic capacities. Sulfate reduction, fermentation, and hydrogen oxidation were predominant while the contributions from aerobic and microaerophilic respiration processes were smaller (Datasheet 1F). In Figure 3, we categorized the gene expression into expression profiles by comparing the coverage values of transcripts for specific functional genes with those from the housekeeping gene *gyrB*. Two Desulfobacterota MAGs—*bin145* (*Desulforhopalus* sp.) and *bin075* (*Desulfobacula* sp.)—expressed *hgc* genes in the redox transition and euxinic zones (BY32 65–90 m, BY15 80–160 m water depths), with values superior or equal to *gyrB* genes. Notably, the *hgc* expression level from *bin075* was high in the redox transition zone of the BY15 station (Figure 3). These two MAGs (*bin075*, *bin145*) also expressed genes involved in sulfate reduction, known metabolic capacities for Desulfobacterota members.^63−65^ with similar or higher coverage values than those of *gyrB* genes; see for instance the strong expression of *bin0145* in the redox transition and euxinic zones at the BY32 station (Figure 3). The Desulfobacterota MAGs, *bin062* (Desulfatiglandales), and *bin036* (Myxococcota UBA796 group) did not express any *hgc* genes but expressed sulfate reduction and fermentation genes at a level similar to the expression of the *gyrB* gene. The two Planctomycetota MAGs *bin278* (Sedimentisphaerales) and *bin009* (Pirellulaes) expressed *hgc* genes in both the redox transition zone and euxinic zone. In accordance with recent findings about their capacity for sulfate reduction,^66^ transcripts of sulfate reduction genes were found for these two Planctomycetota with values higher than *gyrB* genes for *bin009* in euxinic samples (Figure 3). The two *hgc*^*+*^ Verrucomicrobiota MAGs, *bin225* (Kiritimatiellae) and *bin143* (Lentisphaeria) expressed *hgcA* transcripts in euxinic samples (Figure 3) with the highest values at BY32 190 m for *bin143* (Figure 2B). The single Chloroflexota representative (*bin111*, Anaerolineales) expressed *hgc* genes only in the euxinic zone (Figure 3). Although *bin142* (AABM45-125-24 phylum) expressed *hgc* transcripts in the redox transition zone at both stations, they were expressed in the euxinic zone only at BY32. This MAG also had a high expression of genes involved in sulfate reduction and fermentation at the same locations, which is in accordance with the limited knowledge about this phylum.^63^ Overall, differences were found in the *hgc*^+^ MAGs populating the redox transitions and euxinic zones.^67^ These observations suggest that Hg methylators exhibit versatile metabolic capacities^24,25,68^ and that microbial Hg^II^ methylation is not associated with a single metabolism in the Baltic Sea water columns.

### 3.5 Relationships between *hgc* Abundances, Hg^II^ Speciation, and MeHg Formation

**Figure 4:**
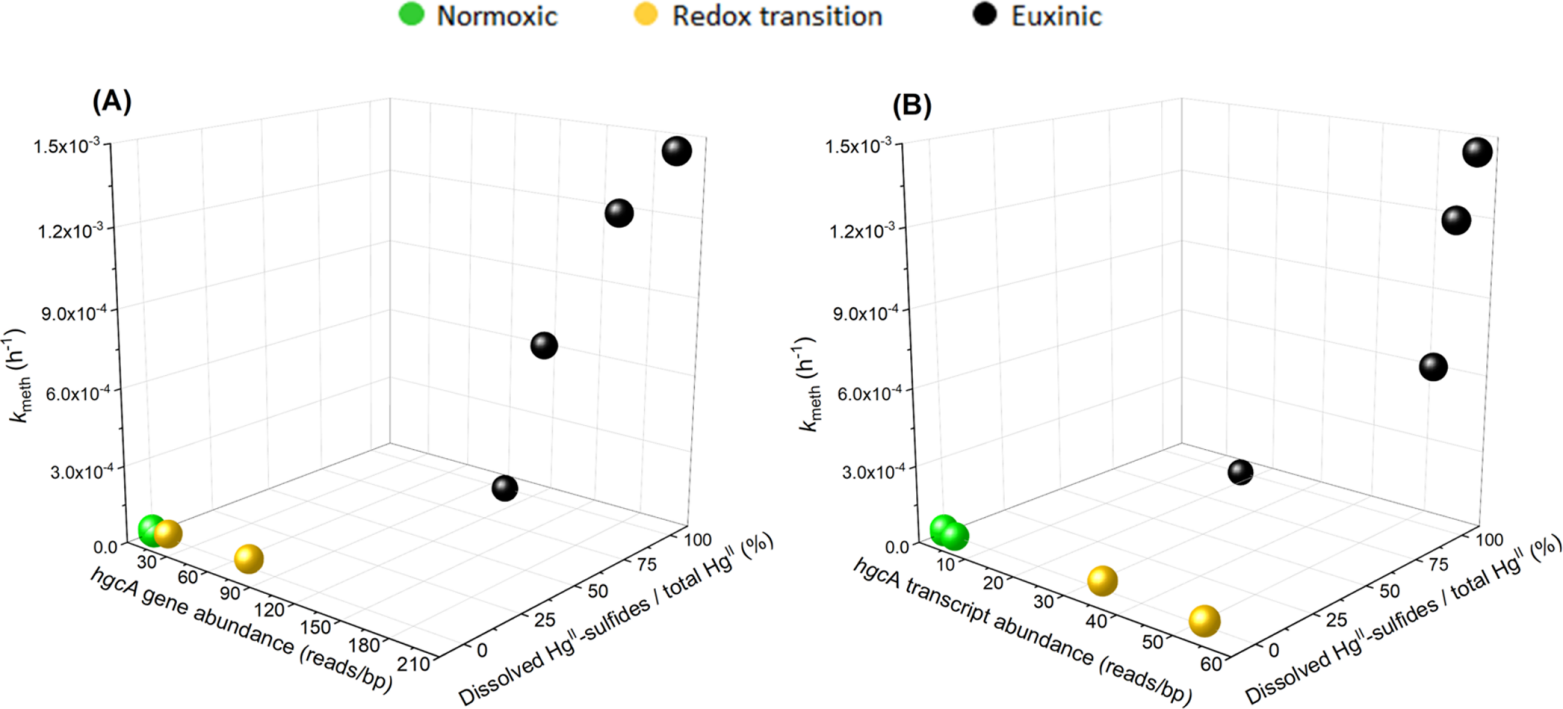
Joint relationship between the *hgcA* gene (A) or transcript (B) abundances (i.e., normalized coverage values as described in Section 2), fraction (% of total Hg^II^) of dissolved Hg^II^-sulfide species and *k*_meth_. Black colored data points correspond to samples from the euxinic zone, with a Hg^II^ speciation dominated by dissolved Hg^II^-sulfide complexes, and yellow and green data points represent samples from the redox transition and normoxic zones, respectively, both void of such complexes.

We investigated potential relationships between both the gene and transcript abundances of *hgc* and the three Hg parameters *k*_meth_, MeHg concentration, and MeHg/HgT molar ratio. There was a correlation between *hgcA* and *hgcB* gene abundances (*r*^2^ = 0.95, *p* < 0.001) and transcript abundances (*r*^2^ = 0.95, *p* < 0.001), and at the gene level the correlations with Hg parameters were significant for both *hgcA* or *hgcB* (Table S3). At the transcript level, however, only *hgcA*—but not *hgcB*—consistently showed significant correlations with all Hg parameters. Except for one study that showed contrasting gene expression levels between *hgcA* and *hgcB* genes for the model strain *Desulfovibrio dechloroacetivorans*,^20^ there is little information about differential expression profiles between these genes and no explanation for such discrepancies. Since the *hgcA* gene is usually used as a microbial marker for Hg^II^ methylation, we focused on this gene for further analysis. Significant positive Spearman rank correlations were found between *k*_meth_ values and the *hgcA* gene (*r*^2^ = 0.58, *p* = 0.048) and transcript abundance (*r*^2^ = 0.65, *p* = 0.023) detected in the water layers from both stations (Figure 4 and Table S3). Low *k*_meth_ (<0.04 × 10^–3^ h^–1^) was found in the redox transition samples and in one euxinic sample (*n* = 3), while high *k*_meth_ (>0.6 × 10^–3^ h^–1^) was found in the other euxinic samples (*n* = 3) (Figure 4). These two groups (*n* = 3 each) contained samples from both stations showing that the grouping in samples with low and high *k*_meth_ was not site-specific. The highest *k*_meth_ was observed for the euxinic samples with the highest *hgcA* gene abundance and expression (Figure 4). We propose that this apparent “threshold” in the relationship between *k*_meth_ and the *hgcA* gene or transcript abundances (i.e., low or nondetectable *k*_meth_ in the redox transition zone despite the significant *hgcA* gene or transcript abundance, Figure 4) was primarily explained by the chemical speciation of Hg^II^. As discussed above, dissolved Hg^II^-sulfide complexes, which have comparably high availability for methylation, dominated the Hg^II^ speciation in euxinic samples (black colored data points in Figure 4). In contrast, adsorbed phases of Hg^II^ with Fe^III^-OM aggregates^30,51^ and the solid-phase HgS(s), with much lower availability for methylation,^53,54^ are expected to dominate the speciation in the redox transition zone (yellow colored data points in Figure 4).

We took into account both *hgcA* abundances and Hg^II^ availability for MeHg formation (Figure 4) by partial least-squares projection (PLS) modeling. Two separate PLS models with the concentration of dissolved Hg^II^-sulfide species and either the *hgcA* gene (*r*^2^ = 0.83, *q*^2^ = 0.62) or transcript (*r*^2^ = 0.69, *q*^2^ = 0.59) abundance as *X*-variables were generated (Table S3). Compared to the models presented above, with *hgcA* abundances only, the fit was improved when taking into account also Hg^II^-sulfide species in the models. The results support the hypothesis that Hg^II^ methylation across the three pelagic redox zones is controlled jointly by the expression of *hgcA* and the concentration of dissolved Hg^II^-sulfide complexes (Figure 4). These two drivers of MeHg formation followed partly different vertical profiles across the redox zones. The *hgcA* genes and transcripts were consistently present and expressed both in the redox transition and euxinic zones, showing that there is a high microbial potential for Hg^II^ methylation in both zones. In the transition zone, however, the rate of Hg^II^ methylation was below or close to the detection limit due to the low availability for methylation of Hg^II^, which most likely was rate limiting for MeHg formation in this zone. In euxinic waters, the availability of Hg^II^ was high throughout the entire zone due to formation of dissolved Hg^II^-sulfide species, and the Hg^II^ methylation rate was limited by the expression of *hgcA* genes.

One euxinic sample (BY15 160 m) exhibited nondetectable *k*_meth_ (Figure 4). The composition and metabolism of the overall prokaryotic community (Figure S7) in this sample, as well as the composition of Hg^II^-methylating groups (Figure 2A), were consistent with other samples from the euxinic waters. However, compared to the samples with high *k*_meth_, the total abundance of *hgcA* transcripts were lower in this sample (Figures 2A and S2), and we suggest that this might explain the low *k*_meth_ values measured for this sample. In this sample, *hgcA* transcripts were associated with four MAGs, including a *Desulfobacula* MAG (*bin075*) and an Anaerolineales MAG (*bin111*). Interestingly, these *hgc*-expressing MAGs contributed less, in proportion, to the expression of genes involved in the metabolic capacities of the overall community (i.e., transcript expression profiles for sulfate reduction and fermentation, respectively) in this sample (0.29 and 0.01%) compared to other euxinic samples (0.78–4.01, 0.03–0.04%) (Datasheet 1D and 1F). This implies that the *hgc*^+^ MAGs could be outcompeted by non-*hgc*^*+*^ MAGs in this layer leading to reduced capabilities of the resident community to methylate Hg^II^.

We evaluated if other processes could explain the apparent threshold in the relationship between *k*_meth_ and *hgcA* genes and transcripts. We considered the central metabolic capacities of microorganisms expressing *hgc* genes and the composition of organic matter (OM) in the water columns. Laboratory experiments with isolated microorganisms have demonstrated large differences in the Hg^II^ methylation capacity within and between microbial phyla,^69^ but it is still not clear if this process is linked to their essential metabolic capacities. Our results showed that there was no systematic difference in metabolic capacities of *hgcA* expressing MAGs in the redox transition and euxinic zones (Figure 3) that could explain the large differences observed in *k*_meth_. Previous studies have demonstrated that the origin and degradation status of organic matter can control Hg^II^ methylation in lakes^70^ and ponds^71^ with contrasting sources of OM. These previous studies used GC-MS and fluorescence spectroscopy (respectively). In our study, we characterized the molecular composition of the dissolved OM (DOM) in all samples using liquid chromatography–electrospray ionization mass spectrometry.^72^ The data obtained show that the DOM composition did not vary within the euxinic zone and differed only marginally between the redox transition and euxinic zones (Supporting Information). Hence, for these water columns it is unlikely that the composition of DOM is an important contributing factor to the observed differences in *k*_meth_.

The *hgcA* gene and transcript abundances were significantly correlated also with the ambient MeHg concentration (*r*^2^ = 0.87, *p* < 0.001, and *r*^2^ = 0.78, *p* < 0.001, respectively) and the MeHg/HgT molar ratio (*r*^2^ = 0.63, *p* = 0.027 and *r*^2^ = 0.58, *p* = 0.048, respectively) (Table S3). Further, like the results for *k*_meth_, PLS models considering both the *hgcA* gene or transcript abundance and concentration of dissolved Hg^II^-sulfide complexes gave a good fit for both the MeHg concentration and the MeHg/HgT molar ratio (*r*^2^ = 0.82, *q*^2^ = 0.70 and *r*^2^ = 0.67, *q*^2^ = 0.40, respectively, for PLS models with the *hgcA* gene abundance and Hg^II^-sulfide complex concentration). In principle, the MeHg concentration and MeHg/HgT ratio are not only determined by *hgcA*-driven Hg^II^ methylation but also by the MeHg demethylation rate and MeHg and Hg^II^ fluxes. It is thus noteworthy that the *hgcA* gene and transcript abundances, together with the concentration of dissolved Hg^II^-sulfide species, could explain most of the variance both in *k*_meth_ determined from an added Hg^II^ isotope tracer in incubation experiments and in the ambient MeHg concentration and MeHg/HgT in the water samples. The tight link between all of the parameters related to MeHg formation shows the importance of methylation of Hg^II^, driven by the *hgcA* gene and transcript abundances and concentration of dissolved Hg^II^-sulfide species, for buildup of the ambient MeHg pool in these redox-stratified waters.

### 3.6 Environmental Implications

The presence of *hgc* genes has proven to be a reliable predictor of Hg^II^ methylation capability in microorganisms.^69,73^ Indeed, deletion of the *hgc* genes impairs Hg^II^ methylation capacity.^8^ Further, Qian et al.^74^ observed an apparent relationship between HgcA protein concentration and the amount of formed MeHg (although no statistical evaluation was reported) in laboratory assays with a sulfate-reducing bacterium. However, no study has demonstrated a quantitative relationship between the expression of *hgc* genes (i.e., *hgc* transcripts) and its associated reaction, (i.e., Hg^II^ methylation rates), neither in environmental samples nor in laboratory culture experiments.^20−22,75^ It has thus remained uncertain if rates of Hg^II^ methylation are constrained by the molecular-level methylation processes mediated by the *hgc* genes. In general, a positive relationship between the abundance of genes or transcripts and the corresponding process rates is often assumed but rarely demonstrated in natural systems, mainly because of a lack of fundamental understanding of the factors that control the activity of the microorganisms and thus the gene transcription.^15^

In this study, we show significant relationships between *hgcA* gene abundance as well as *hgcA* gene transcripts and all three quantified MeHg parameters *k*_meth_, MeHg/HgT molar ratio, and MeHg concentration. Our results thus support that the rate of Hg^II^ methylation and the accumulation of MeHg in redox-stratified water is constrained by the expression of the *hgc* genes. Our results further show that models with both *hgcA* genes or transcript abundances and Hg^II^ chemical speciation improved the prediction of Hg^II^ methylation rates in redox-stratified pelagic waters, suggesting that *hgcA* and Hg^II^ availability jointly control MeHg formation in such environments. These key findings are conceptually illustrated in the graphical abstract. The observed presence of *hgcA* genes and transcripts in the redox transition zone indicates that there is microbial potential for Hg^II^ methylation under such conditions. The chemical speciation and availability for methylation of Hg^II^ are, however, expected to fluctuate substantially even with moderate variations in H_2_S concentrations in this zone. As a consequence, large spatial and temporal variability in the Hg^II^ methylation rate, driven by H_2_S concentrations, may be expected. In contrast, Hg^II^ availability for methylation is high throughout the euxinic water zone and the Hg^II^ methylation rate is instead controlled by the abundance and expression of *hgcA* genes.

The native function of the *hgc* genes is still not known,^8,76^ but they do not seem to be part of a Hg detoxification system since the gene expression is not induced by Hg exposure and does not confer Hg resistance.^20,75^ Our results suggest that Hg^II^ methylation is, to some extent, associated with the overall microbial activity of certain microorganisms as well as their specific metabolic capacities such as sulfate reduction, fermentation, and hydrogen oxidation (Figures 3A and S7). Overall, our results point to these three principal metabolisms among putative Hg^II^ methylators in the redox-stratified water column of the central Baltic Sea. We hypothesize that, without having an evident causality link between Hg^II^ methylation and other metabolic processes, the characterization of the biogeochemistry and associated metabolism of microbial communities inhabiting aquatic systems is a novel and useful approach to better predict the presence of Hg^II^ methylation in aquatic systems.

Based on high observed MeHg concentrations in oxygen-deficient environments and the fact that the vast majority of known microorganisms capable of Hg^II^ methylation are anaerobes, it is expected that the current and future spread of oxygen deficiency in coastal seas and the global ocean will increase Hg^II^ methylation rates and likely MeHg concentrations. However, quantitative predictions have been hampered by a lack of understanding of principle drivers controlling Hg^II^ methylation under such conditions. The results of our study benchmark the use of *hgcA* genes and transcripts and their abundances as strong predictors, together with dissolved Hg^II^ sulfide species, for Hg^II^ methylation in redox-stratified waters. The unraveling of these mechanistic principles governing Hg^II^ methylation and MeHg concentrations in oxygen-deficient coastal seas is a critical step for refining predictive frameworks for MeHg exposure to marine food webs and humans from the current and future spread of oxygen deficiency in coastal waters and the global ocean, as well as for MeHg formation in the environment in general.

## Supporting Information Available

This file includes descriptions, figures, and tables about the Hg speciation and PLS models used; DOM composition of the studied water samples; description of molecular analysis and bioinformatics methods, the composition and metabolism of the prokaryotic community; and the correlation values between biological data and Hg data (PDF)
Information related to different parameters collected or measured in this work from samples collected at stations BY32 and BY15 **(**A) Data related to water depth profiles of ancillary parameters (temperatures, salinity, oxygen, hydrogen sulfide), HgT, MeHg concentrations, the MeHg/HgT molar ratio (%), and Hg^II^ methylation rate constant (*k*_meth_), and *hgc* gene and transcript abundances (normalized coverage values as described in the material and methods); (B) DNA and RNA concentrations measured by Qubit and Nanodrop, number of raw and trimmed reads, mean coverage values of the housekeeping gene *gyrB*; (C) data related to each detected *hgc* genes, their amino acid sequences, abundance metrics, taxonomic identification and bins id; (D) abundance data of the 39 functional genes extracted from the metagenomes and metatranscriptomes; (E) data related to the reconstruction of good-quality MAGs from metagenomes; (F) coverage values of the 39 functional genes retrieved in the 10 *hgc*^+^ MAGs normalized by *gyrB* coverage values; and (G) coverage values of *gyrB* and all functional genes retrieved in the 10 *hgc*^+^ MAGs (Datasheet 1) (XLSX)
