# Supplementary material for: Expression Levels of hgcAB Genes and Mercury Availability Jointly Explain Methylmercury Formation in Stratified Brackish Waters
Source: Environ Sci Technol. 2022 Sep 7;56(18):13119–30. doi: 10.1021/acs.est.2c03784 (PMC9494745; doi:10.1021/acs.est.2c03784)
Supplement: Supplementary file 1 — es2c03784_si_001.pdf [file es2c03784_si_001.pdf]

Supplementary information

Expression levels of *hgcAB* genes and mercury availability jointly explain methylmercury formation in stratified brackish waters

Eric Capo[1,2,a], Caiyan Feng[1,a], Andrea G. Bravo[3], Stefan Bertilsson[2], Anne L. Soerensen[4], Jarone Pinhassi[5], Moritz Buck[2], Camilla Karlsson[5], Jeffrey Hawkes[6] , Erik Björn[1,*]

[a]Joint first authors

*Corresponding author: erik.bjorn@umu.se

[1]Department of Chemistry, Umeå University, Umeå, 901 87, Sweden
[2]Department of Aquatic Sciences and Assessment, Swedish University of Agricultural Sciences, Uppsala, 75007, Sweden
[3]Department of Marine Biology and Oceanography, Institute of Marine Sciences, Spanish National Research Council (CSIC), Barcelona, 08003, Spain
[4]Department of Environmental Research and Monitoring, Swedish Museum of Natural History, Stockholm, 104 05, Sweden
[5]Centre for Ecology and Evolution in Microbial Model Systems - EEMiS, Linnaeus University, Kalmar, 391 82, Sweden
[6]Department of Chemistry, Uppsala University, Uppsala, 75123, Sweden

Number of pages: 16
Number of figures: 9
Number of tables: 3

<u>Study site – Baltic Sea</u>

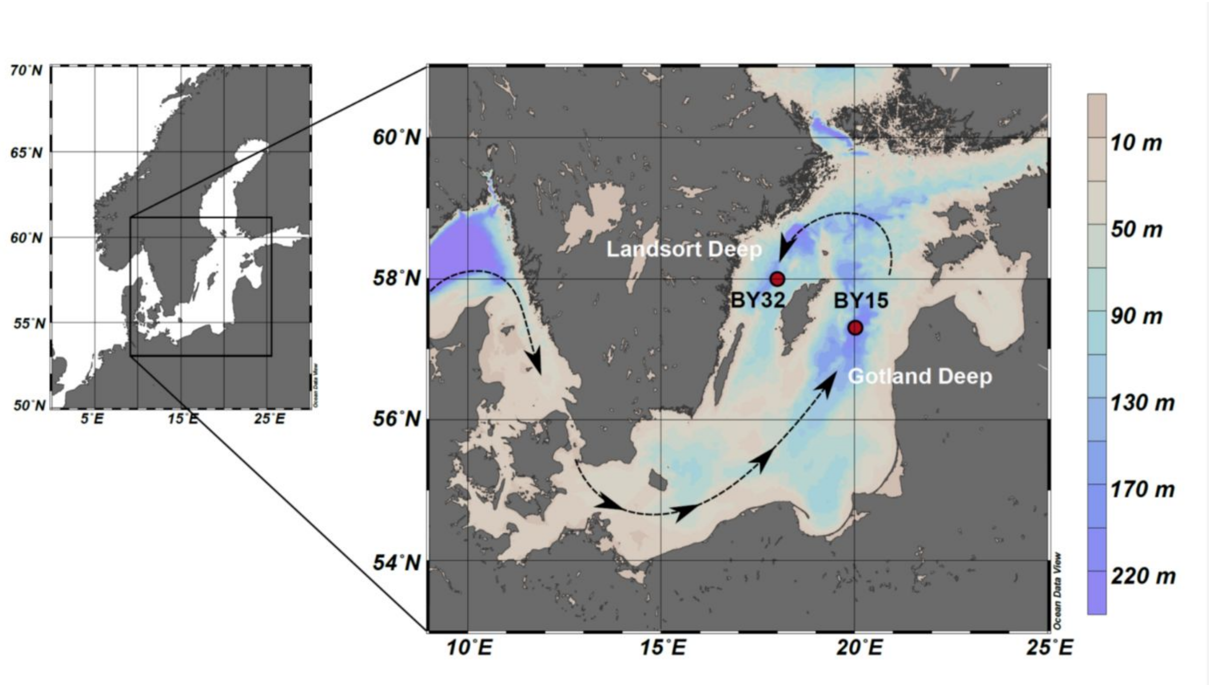

Figure S1. Location of the stations BY32 in the Landsort Deep and BY15 in the Gotland Deep in the central Baltic Sea. Black arrows show the entry point and primary circulation of saline North Sea water below the halocline relevant for this study.

Speciation model

The chemical speciation of Hg^{II} and MeHg was determined by thermodynamic modeling using the Solgaswater (WinSGW) software (Karlsson & Lindgren, 2013). We used Hg-species and stability constants from the model by Liem-Nguyen et al., ES&T 2017, but adjusted pH, ionic strength and the concertation of all components to fit the Baltic Sea environment model matrix is given in Table S1 and the input concentrations and modeling output for Hg^{II} for each sample is given in Table S2. The pH and Cl^- concentration were fixed at 7.3 and 90 mM, respectively for all the samples. The concentration of thiols associated with DOM (DOM-RSH) were calculated as 0.15% mass fraction DOM-RSH of the DOC concentration (Skyllberg *et al.* 2006). The DOC concentration was measured at all 6 depths at BY32 and at 6 of the 12 depths at BY15. A second order polynomial function was fitted to calculate a DOC concentration at the remaining depths. MeHg species were included in the model for completion but are not further discussed.

Table S1. Reactions and logarithmic thermodynamic formation constants (log K_f) for Hg^{II} and MeHg species included in the speciation models.

		Components					
**Species**	**Log K_f**	**H^+**	**Cl^-**	**DOM-RS$^-$**	**H_2S**	**Hg^{2+}**	**$MeHg^+$**
**OH^-**	-13.7	-1					
**HS^-**	-7	-1			1		
**DOM-RSH**	9	1		1			
**$HgOH^+$**	-3.4	-1				1	
**$Hg(OH)_2$**	-6.2	-2				1	
**$HgCl^+$**	7.1		1			1	
**HgCl2**	13.8		2			1	
**$HgCl_3^-$**	14.7		3			1	
**$HgCl_4^-$**	15.4		4			1	
**HgOHCl**	7.8	-1	1			1	
**$Hg(DOM\text{-}RS)_2$**	41			2		1	
**$HgSH^+$**	13.72	-1			1	1	
**HgS_2H^-**	18.1	-3			2	1	
**HgS_2^{2-}**	9	-4			2	1	
**$Hg(SH)_2$**	24.6	-2			2	1	
**HgClSH**	18.89	-1	1		1	1	
**HgOHSH**	9.42	-2			1	1	
**HgS(s)**	30.3	-2			1	1	
**MeHgOH**	-4.5	-1					1
**MeHgCl**	5.4		1				1
**MeHg(DOM-RS)**	16.5			1			1
**MeHgSH**	7.62	-1			1		1
**$MeHgS^-$**	0.12	-2			1		1
**$S(MeHg)_2$**	16.42	-2			1		2

Table S2. Input concentrations and speciation modeling output (% distribution of major species) for all water samples.

		Input parameters				Model output			
	Depth	Hg^{II}	MeHg	H_2S	DOM-RSH	$Hg(SH)_2$	HgS_2H^-	HgS_2^{2-}	$Hg(DOM\text{-}RS)_2$
**BY15**	**(m)**	**(fM)**	**(fM)**	**(µM)**	**(µM)**	**(%)**	**(%)**	**(%)**	**(%)**
Normoxic	5	851	49	0	0.174	0	0	0	100
	25	401	49	0	0.164	0	0	0	100
	40	701	49	0	0.158	0	0	0	100
	60	651	49	0	0.15	0	0	0	100
	65	419	81	0	0.148	0	0	0	100
	70	801	49	0	0.147	0	0	0	100
	75	501	49	0	0.145	0	0	0	100
Transition	80	951	49	0	0.143	0	0	0	100
	130	973	227	0	0.133	0	0	0	100
Euxinic	160	2250	950	22	0.127	10.1	86.5	3.4	<0.001
	195	446	1454	44	0.125	10.1	86.5	3.4	<0.001
	230	790	1510	100	0.126	10.1	86.5	3.4	<0.001
**BY32**									
Normoxic	25	351	49	0	0.173	0	0	0	100
	55	451	49	0	0.159	0	0	0	100
	65	501	49	0	0.155	0	0	0	100
Transition	75	691	859	0	0.152	0	0	0	100
Euxinic	90	275	1275	30	0.148	10.1	86.5	3.4	<0.001
	190	1033	867	41	0.141	10.1	86.5	3.4	<0.001

PLS model

Partial least squares (PLS) projection models were generated in the Simca 15 (Sartorius Stedim Data Analytics AB) software. Separate models (N=12 for each model) were generated with either the *hgcA* gene or transcript abundances as quantitative x-variables. In all models, the concentration of dissolved Hg^{II}-sulfide complexes (sum of $Hg(SH)_2$, HgS_2H^- and HgS_2^{2-}) were used as a qualitative x-variable (0 or 100%) based on the result of the speciation modeling (Table S2). X-variables were centered and scaled to unit variance. Separate models were generated with k_{meth}, MeHg concentration or MeHg/HgT molar ratio as y-variable. For each model, two PLS components were fitted. The generated models were thus equivalent to multiple linear regression models with each model consisting of two x-variables and one y-variable. The Simca software and PLS models were used for technical reasons.

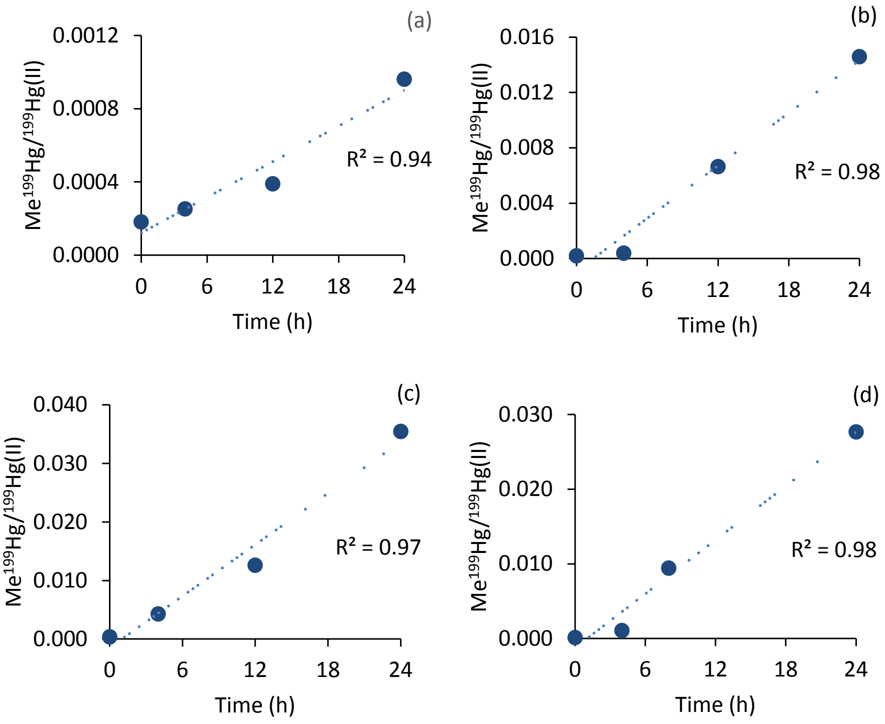

Figure S2. Formation of $Me^{199}Hg$ from added $^{199}Hg(II)$ (expressed as $Me^{199}Hg/^{199}Hg(II)$ molar ratio) over time in water sample incubation experiments for sample with a significant Hg(II) methylation rate constant (k_{meth}), i.e. samples (a) BY32 75m, (b) BY32 90m, (c) BY32 190m and (d) BY15 230 m.

The DOM composition of the redox gradient from the Central Baltic Sea is stable

In this first DOM composition data interpretation, there was one clear outlier (Bal-BY32-25m). The pair of Suwannee River Fulvic Acid reference material analyses gave a dissimilarity of 2.9%, indicating high method precision. The rest of the Baltic samples were highly similar to each other, with dissimilarity values generally <10%. Fig. S6 shows a heatmap of the Baltic samples alone, without the outlier. Overall, the molecular composition of DOM was found to be very similar at both stations and at all water depths. In particular, no pattern was found with depth (e.g., above and below the redox boundaries), indicating that compositional differences in ionizable DOM molecules do not explain differences in Hg species abundances or formation potential. Rather, our data support that these parameters are controlled by the presence of inorganic sulfide species and *hgc*-carrying microorganisms.

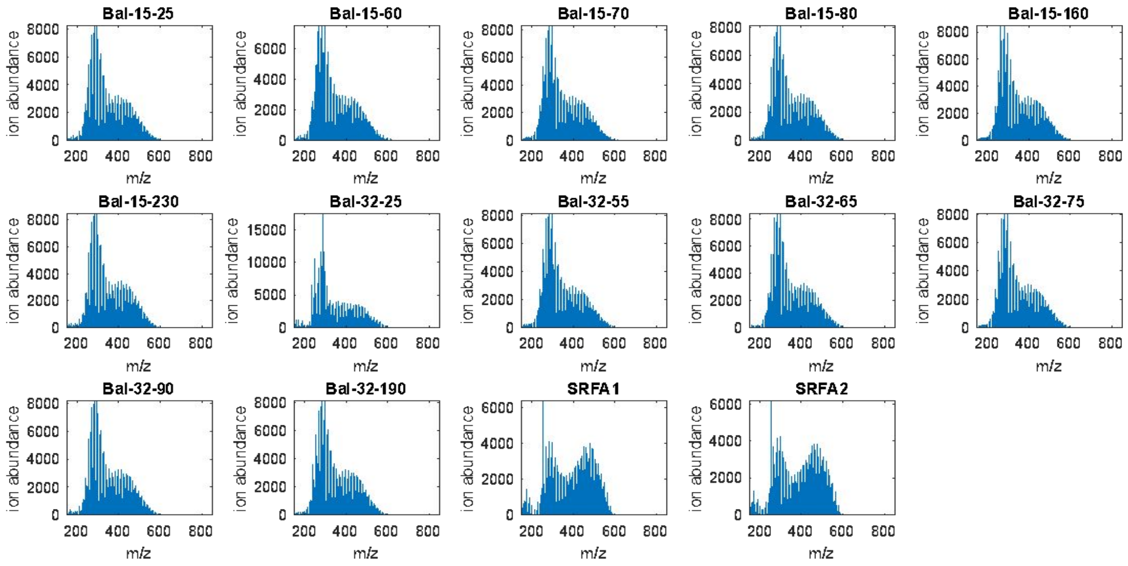

Figure S3. Mass spectra of the extracted DOM samples.

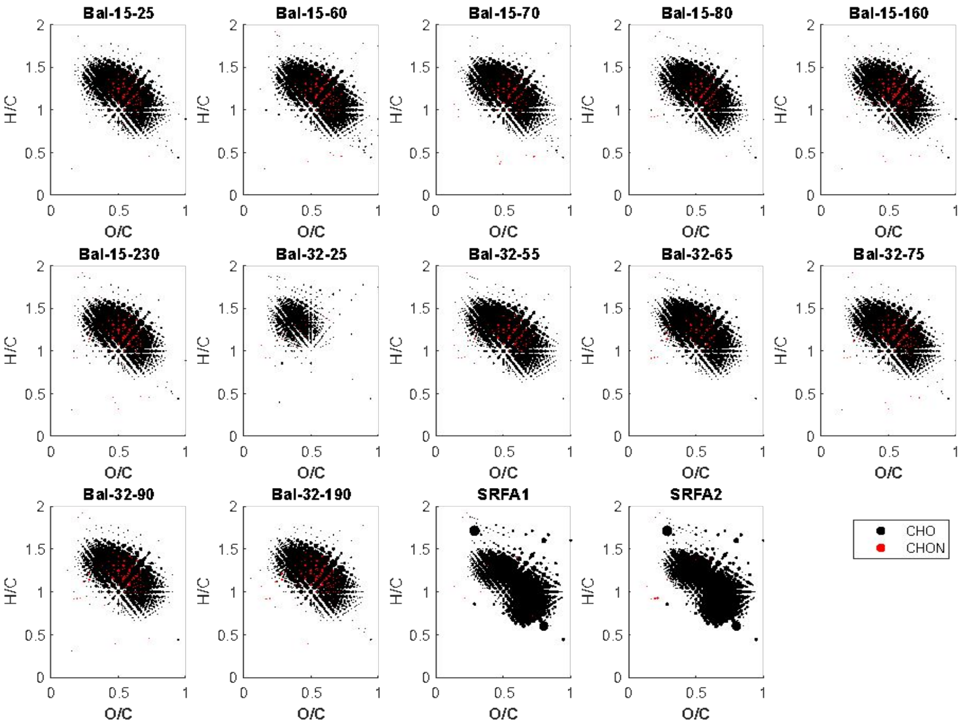

Figure S4. van Krevelen diagrams (H/C vs O/C ratio) of assigned formulas for the extracted DOM samples. Point size indicates summed peak intensity.

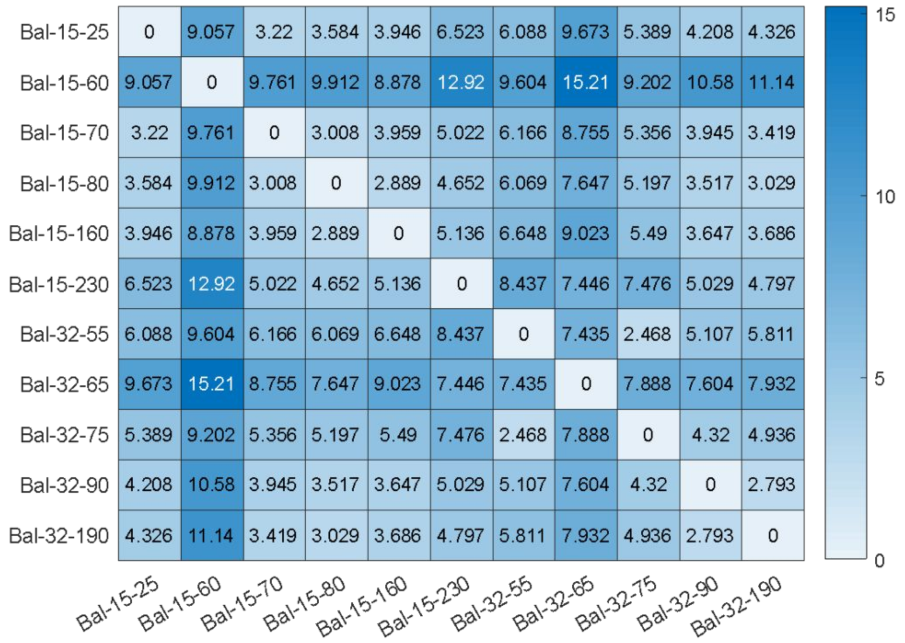

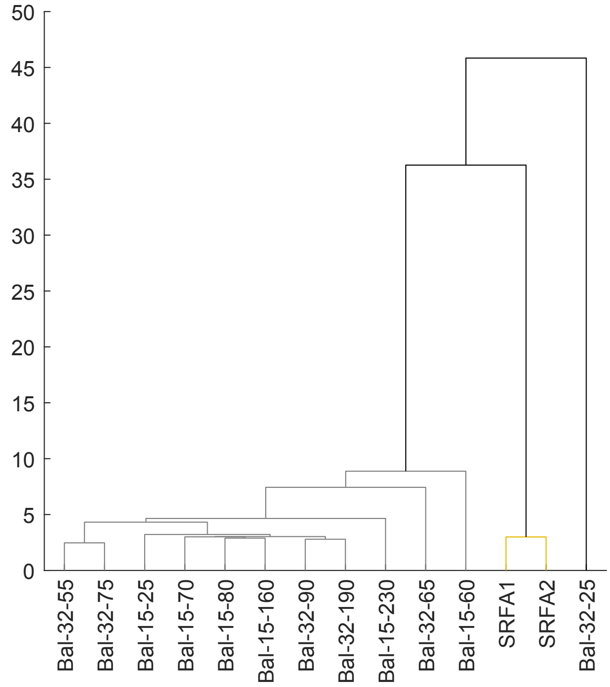

Figure S5. (A) Heatmap of Bray Curtis Dissimilarities for the molecular composition of the extracted DOM samples. (B) Hierarchal Cluster Analysis of Bray Curtis Dissimilarities.

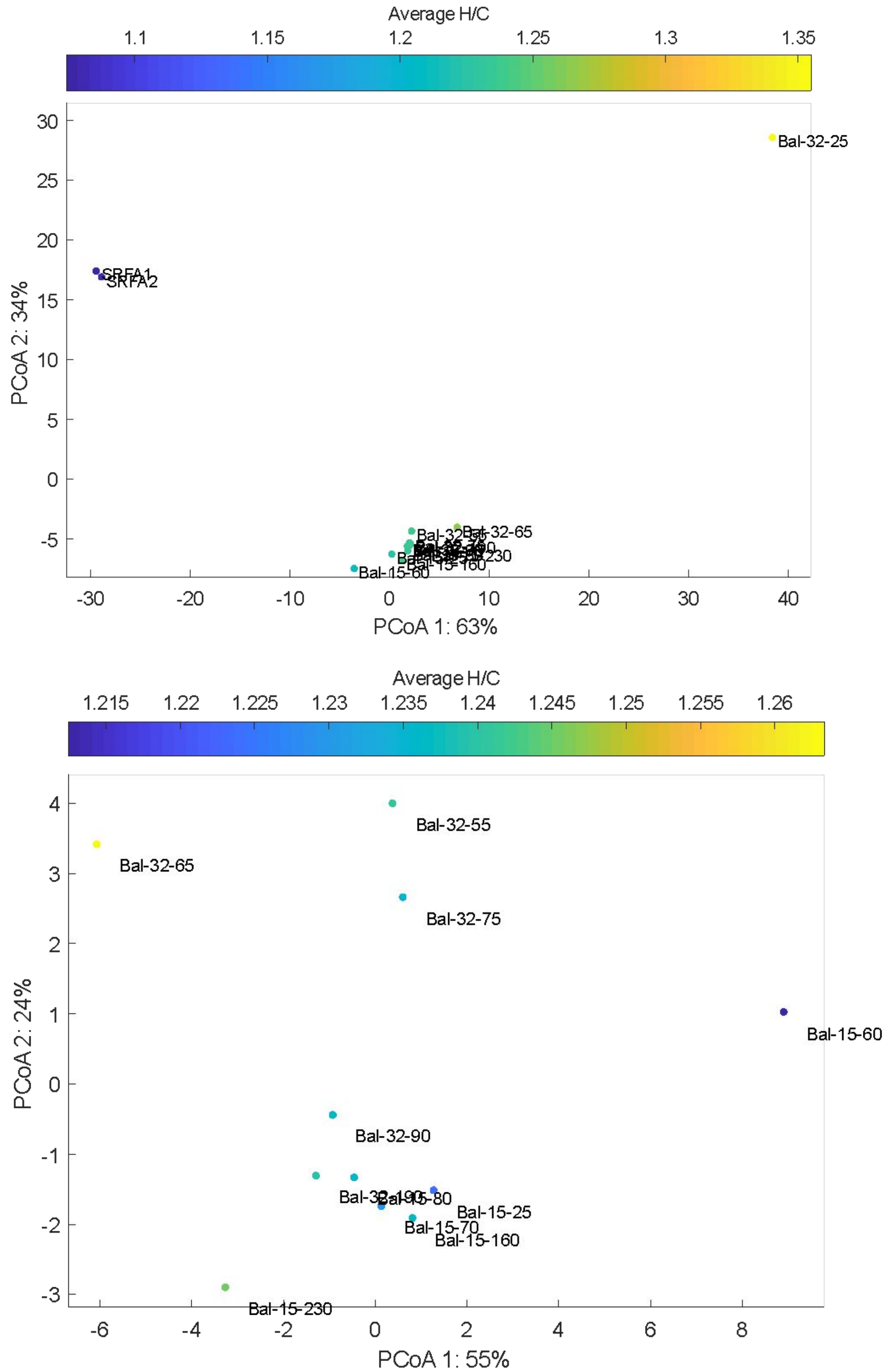

Figure S6. The top figure shows the principal coordinate analysis of Bray Curtis Dissimilarities for all sample, indicating high dissimilarity between the majority of samples and site 32 at 25m, and also the reference mixture of SRFA. The bottom figure shows the principal coordinate diagram of non-outlying samples.

Molecular analysis and bioinformatics

Trimmomatic v0.36 was used to quality trim the data with the following parameters: LEADING:5 TRAILING:5 MINLEN:36 ILLUMINACLIP:TruSeq3-PE-2.fa:2:30:10:2 (Bolger *et al.* 2014). The final quality was validated using FastQC v0.11.5 (Andrews 2010). The metagenome assembly of the DNA samples, generated using the assembler MEGAHIT v1.1.2 (Li *et al.* 2015) with default settings, yielded 3,859,129 contigs. The DNA and RNA reads were mapped against the contigs with the function bowtie2-build and bowtie2 from the software bowtie2 (Langmead & Salzberg 2012) and the resulting .sam files were converted to .bam files using the function *samtools* in the software samtools v1.9 (Li *et al.* 2009). On average 26,954,264 DNA sequences and 11.396.571 RNA sequences were mapped onto the assembly (Datasheet 1B). This was followed by annotation of the contigs with the *prokka* function in Prokka v1.12 (Seemann 2014) that uses Prodigal v2.6.3 (Hyatt *et al.* 2010) for prokaryotic gene prediction and BLAST v2.6.0+ (Altschul *et al.* 1990) for sequence alignment of translated nucleotides. Prokka was run with a metagenome setup against the UniProtKB/Swiss-Prot protein database (Release18Feb2020) with the following settings: --proteins uniprot_sprot.fasta --metagenome. The .bam files and the prodigal output .gff file were used to estimate sequence counts by using the function *featureCounts* in the software Subread v1.5.1 (Liao *et al.* 2014).

Bins were generated using the function *metabat2* of the software Metabat2 v2.12.1 (Kang *et al.* 2019) (Datasheet 1E). The function *checkM* from the software checkM v1.1.2 (Parks *et al.* 2015) was used to estimate the completeness and redundancy of each bin with the following settings: -- taxonomy_wf life Prokaryote. The software GTDB-Tk v0.3.2 (Chaumeil *et al.* 2019) was used with the following settings: -- classify_wf to taxonomically classify each bin. To annotate the contigs and calculate their coverage in each bin, the functions *prokka* (Prokka v1.12 (Seemann 2014) and *jgi_summarize_bam_contig_depths* (Metabat2 2.12.1 Kang *et al.* 2019) were used. Out of the 280 bins generated in this study (Datasheet 1E), 50, 77 and 121 were respectively defined as high-quality draft (>90% complete, <5% contamination), medium-quality draft (>50% complete, <10% contamination) or low-quality draft (<50% complete, <10% contamination) MAGs respectively. Finally, 32 bins had no reported completeness and contamination values and were not even classified. In the present work, we referred to MAGs only for high-quality and medium-quality MAGs, the low-quality MAGs being not considered as true signals. Altogether 127 good-quality MAGs were reconstructed from the 12 metagenomes.

The metabolic capacity of overall microbial community and of specific MAGs was evaluated using the hidden Markov models (HMMs) of Pfam (Finn et al. 2010) and TIGRFAM (Selengut et al. 2007) databases for 39 functional genes (Datasheet 1D) and applying the function *hmmsearch* from the software hmmer v3.2.1 (Finn et al. 2011). Verified hits of the 39 functional genes were detected using the trusted cut-off provided in each HMM file. For each sample and each functional gene, coverage values (number of reads per base) (see Datasheet 1D). The housekeeping gene *gyrB* was detected using the HMM profiles TIGR01059.hmm and applying the trusted cut-off provided in HMM files. The overall community composition was evaluated using the *kraken2* function from kraken2 (Wood et al. 2019) with default settings (v2.0.8) for the taxonomic classification of the sequences obtained in the metagenomes and metatranscriptomes.

Detection, taxonomic identification and normalization of *hgc* genes and transcript

We first looked for *hgc* gene homologs in the 5,433,642 predicted protein coding genes with the function *hmmsearch* from hmmer (Finn et al. 2011) software (v3.2.1) and using the HMM profiles provided by Hg-MATE database (v1.01142021, doi:10.25573/serc.13105370) built from multiple sequences alignments of *hgcA* and *hgcB* concatenated amino acid sequences. We considered genes with E-values < 10^{-3} as significant hits resulting in 5,174 hits. To identify which of the hit genes truly correspond to *hgcA* and *hgcB* genes, we used the knowledge from the seminal paper of Parks et al. (2013) and Smith et al. (2015) that described unique motifs from *hgcA* (NVWCA(A/G/S)GK) and *hgcB* genes (C(M/I)EC(G/S)(A/G)C) and performed a manual check of the presence of *hgcA* and *hgcB* genes in our dataset resulting in 126 confirmed *hgc* genes, 77 *hgcA* and 49 *hgcB* genes being identified. In this work, we called the detected genes *hgc* genes; however, because their role in Hg methylation could not be verified solely based on environmental genomics data, they are actually defined as putative *hgc* genes. In some cases, *hgcA* and *hgcB* genes were found side-by-side on the same contig. Overall, we detected 23 *hgcAB* gene pairs and 54 *hgcA* genes. The *hgc* genes reads that were detected in sampling and extraction control metagenomes were considered as absent from samples in they account for less than 6 reads as it is the maximum number of reads of *hgc* genes found in control metagenomes. Coverage values of each hgc gene and transcripts were calculated as the number of reads mapped to each gene divided by the number of bases (reads/bp). Genes and transcripts coverage values were normalized by dividing them with the mean coverage values of the housekeeping gene *gyrB* (Datasheet 1B).

To taxonomically identify hgc-carrying microorganisms detected in BY32 and BY15 samples, we coupled information obtained from *hgc* phylogeny and metagenome-assembled genomes (MAG) that carried *hgc* genes. For *hgc* phylogeny, we used the reference package 'hgcA' and 'hgcAB' from the recent database Hg-MATE v1.01142021 (Gionfriddo et al. 2021) that compiled a total of 1020 *hgc* sequences from publicly available isolate genomes (n =204), single-cell genomes (n=29) and metagenome-assembled genomes (n=787). Briefly, amino acid sequences from gene previously identified as hgcA gene were (i) compiled in a FASTA file, (ii) aligned to Stockholm formatted alignment of HgcA amino acid sequences from the reference package with the function *hmmalign* from hmmer software v3.2.1 (Finn *et al.* 2011), (iii) placed onto the HgcA reference tree with the function *pplacer* and (iv) classified using the functions *rppr* and *guppy_classify* from the program pplacer (Masten *et al.* 2010). For more details, see in the README.txt of Hg-MATE v1.01142021 (Gionfriddo *et al.* 2021). Among the 127 good-quality MAGs obtained in the present study, ten featured *hgc* genes in their genomes hereafter described as *hgc*-carrying MAGs (Fig. S7, Datasheet 1E). Eight of the *hgc*-carrying MAGs carried *hgc* gene pairs while two had only *hgcA* genes in their genomes (*bin036* ad *bin143*). One MAG, *bin225*, included two *hgc* gene pairs and the strain heterogeneity of this MAG was 100 % (Datasheet 1E) indicating that it likely represents multiple genomes. The 10 *hgc*-carrying MAGs were taxonomically identified as members of Desulfobacterota (1 desulfobacteral *Desulfobacula*, 1 desulfobulbal *Desulforhopalus*, 1 desulfatiglandal NaphS2), PVC superphylum (2 Planctomycetes, 1 Kiritimatiellae, 1 Lentisphaerae), and three other microbial phyla (Chloroflexota, Myxococcota and AABM12-125-24) (Fig. S7). The remaining *hgc* genes were affiliated to Desulfobacterota (n=43), PVC superphylum (n=27), Chloroflexota (n=9) and various bacterial and archaeal lineages (3 Bacteroidetes, 1 Spirochaetes, 1 Firmicutes, 1 Elusimicrobia, 1 Thermoplasmatota) and unidentified microorganisms (n=20) (Datasheet 1C).

The composition and metabolism of the prokaryotic community from the Central Baltic Sea is linked to redox conditions of its oxygen-deficient water column

The exploration of the metagenomes and metatranscriptomes obtained from the Landsort Deep and the Gotland Deep water columns allowed us to explore the composition and activity of their resident prokaryotic communities. Overall, a total of 127 good-quality metagenome-assembled genomes (MAGs) were recovered from the water column of the two studied stations (BY32 and BY15) (Fig. S7C, Datasheet 1E). High numbers of MAGs were identified as Actinobacteria (n=21), Gammaproteobacteria (n=12), Verrucomicrobia (n=12) and Planctomycetes (n=11). Additionally, the abundance (summed coverage values) of these 56 MAGs accounted for 60 % of the overall prokaryotic community although they accounted for lower transcript abundance (27 %). At both stations, transcripts from Synechococcales (Cyanobacteria) and photosynthesis genes were predominant in the normoxic zone (25 m) (Fig. S7D & S7B) in agreement with previous knowledge about the repeated occurrences of *Synechococcus* in the water from the Central Baltic Sea (Stal *et al.* 2003). The underlying redox transition zone exhibited increased amounts of transcripts from Thermoproteota (Fig. S7A) and more specifically the MAG *bin123* (*Nitrosopumilus* sp., Nitrososphaerales) (Datasheet 1E). Consistently, members of this obligately aerobic nitrifying archaeal genus have been previously reported in oxygen-deficient waters in the central Baltic Sea (Labrenz *et al.* 2010, Feike *et al.* 2011). Additionally, transcripts from Campylobacterota were detected as highly abundant in the deepest euxinic zones (Fig. S7A). These transcripts were expressed by *Sulfurimonas* sp., (3 dominant MAGs, Datasheet 1E), which are bacteria known to perform denitrification and sulfide oxidation in oxygen-deficient waters including in the Baltic Sea, e.g., (Grote *et al.* 2008, Bergen *et al.* 2018, Beier *et al.* 2019). Their occurrence was coupled with increased transcripts of denitrification and sulfide oxidation genes peaking in both redox transition zones (75 and 80 m at BY32 and BY15 stations, respectively). Although these metabolic functional genes were less abundant in deeper samples, Campylobacterales transcripts remain abundant in underlying water layers supporting knowledge that these bacteria can potentially exhibit metabolic versatility (Grote *et al.* 2008, Beier *et al.* 2019). Finally, Desulfobacterota, known to include numerous sulfate-reducing bacteria (Muyzer & Stams 2008), were more abundant in the euxinic zone (Fig. S7A) in association with increased abundance of transcripts from sulfate reduction genes (Fig. S7B) and hydrogen sulfide concentrations (Fig. 1) in line with previous knowledge about the role of sulfate-reducing Desulfobacterota in the metabolism of oxygen-deficient water columns Canfield *et al.* 2010, Van Vliet *et al.* 2020).

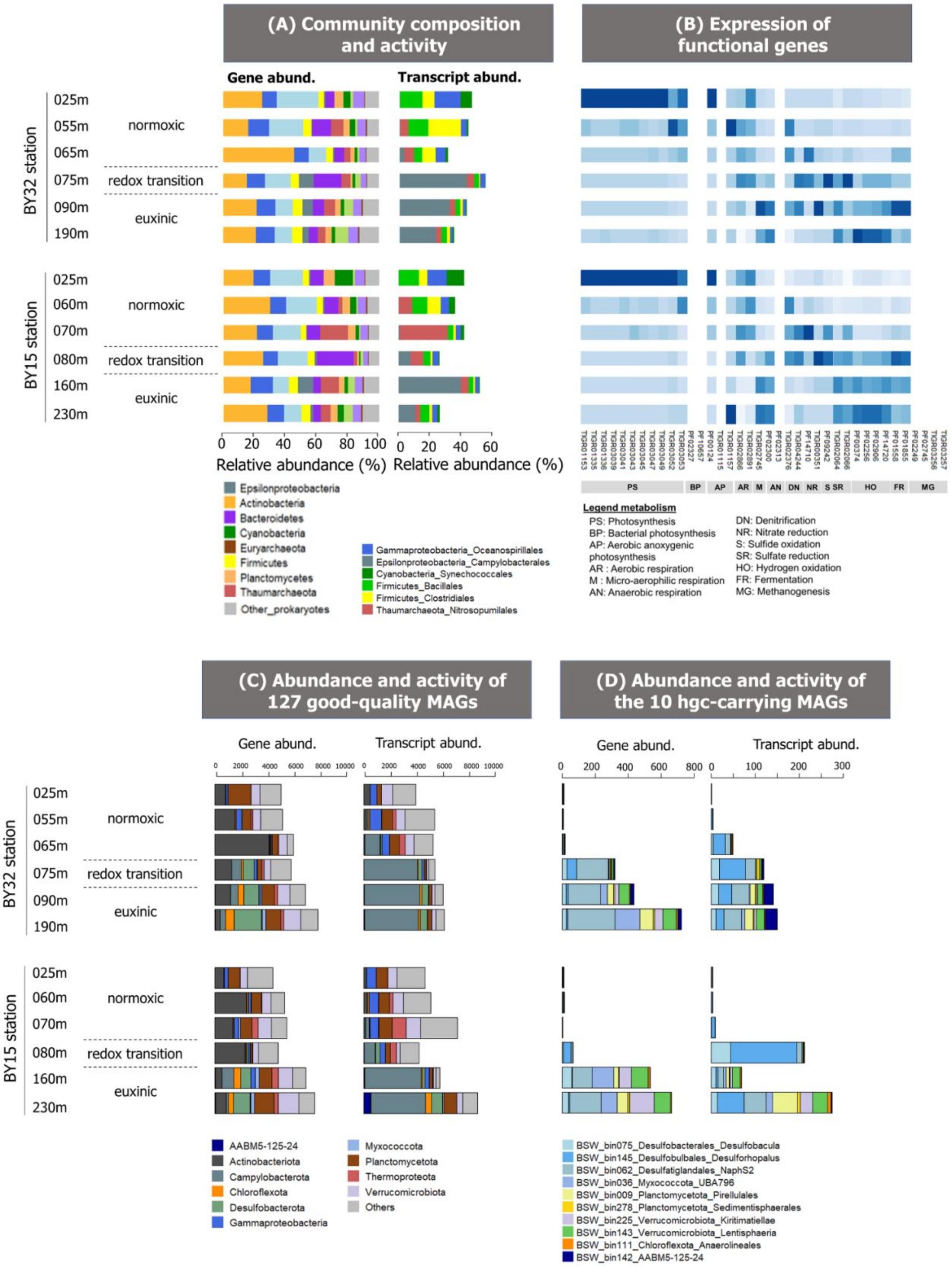

Figure S7. (A) Community composition and activity (B) Heatmap showing the z-scored expression level of each of the 38 functional genes analyzed in the present study covering a broad spectrum of microbial metabolism (e.g., photosynthesis, sulfur cycle and fermentation) Columns that are empty corresponds to transcripts of functional genes not detected in the metatranscriptomes. (C) Representation of MAGs in terms of normalized total gene and transcript coverage values (D) Representation of the 10 hgc-carrying MAGs in terms of normalized total gene and transcript coverage values. Color codes correspond to the taxonomic identification of each microbial group.

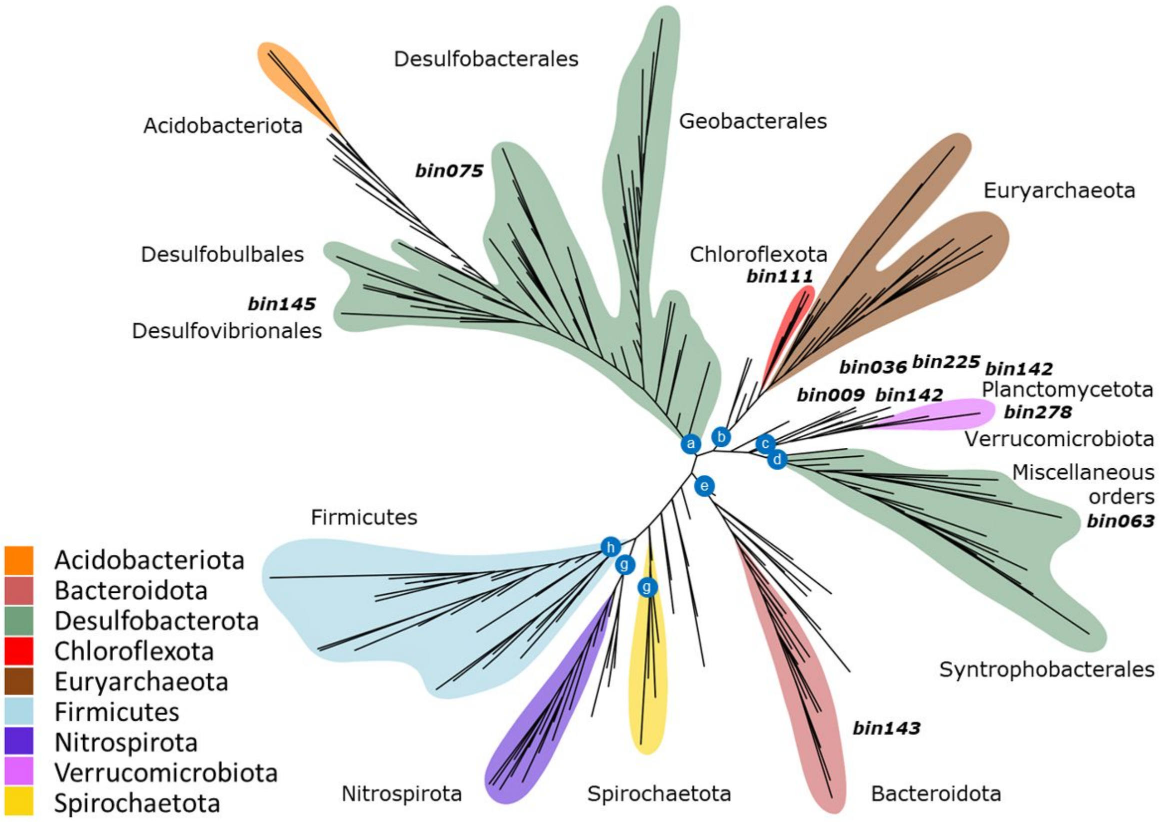

Figure S8. HgcA phylogenetic tree showing the diversity of putative Hg methylators found in the metagenomes obtained from Baltic Sea water column. Binned hgcA genes are denoted in the figure by their id number; unbinned hgcA genes are clustered within groups a,b,c,d,e,g & h (see in Datasheet 1C for correspondence).

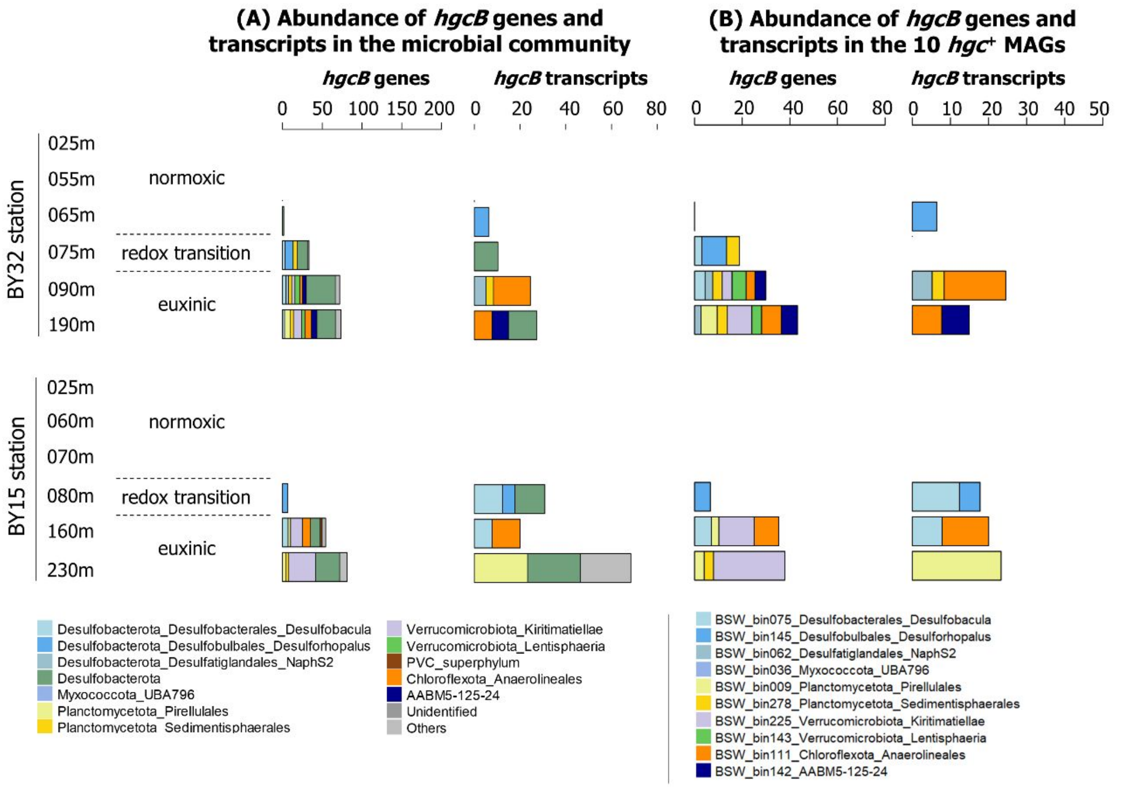

Figure S9: Distribution of *hgcB* genes in terms of abundance (coverage values in read/bp normalized with mean coverage values from the housekeeping gene *gyrB*) of both total genes and transcripts for all *hgcB* genes detected in the water metagenomes (A) and the 10 *hgc*-carrying MAGs (B). Color codes correspond to the taxonomic identification of each microbial group, see bottom panels for correspondence.

Table S3. Spearman rank correlations calculated between selected environmental and biological parameters from the overall prokaryotic community. Abundance was calculated as coverage values (reads/bp) normalized by the coverage values of the housekeeping *gyrB*. The r values denote Spearman's rho rank correlation coefficients, p denotes p-values.

Parameters	[MeHg]		[MeHg]/[Hg]		k_{meth}		overall *hgcA* gene abundance		overall *hgcB* gene abundance		overall *hgcA* transcripts abundance	
	r	p	r	p	r	p	r	p	r	p	r	p
**[MeHg]/[Hg]**	0.86	0.000										
**km rates**	0.62	0.031	0.76	0.004								
**overall *hgcA* gene abundance**	0.87	0.000	0.63	0.027	0.58	0.048						
**overall *hgcB* gene abundance**	0.91	0.000	0.70	0.012	0.64	0.025	0.95	0.000				
**overall *hgcA* transcript abundance**	0.78	0.003	0.58	0.048	0.65	0.023	0.93	0.000	0.96	0.000		
**overall *hgcB* transcript abundance**	0.73	0.007	0.44	0.150	0.47	0.120	0.87	0.000	0.92	0.000	0.95	0.000

References for Supporting Information

Altschul SF, Gish W, Miller W, Myers EW, Lipman DJ. Basic local alignment search tool. J Mol Biol 1990; 215: 403–410. Andrews S FastQC: a quality control tool for high throughput sequence data

Beier S, Holtermann PL, Numberger D, Schott T, Umlauf L, Jürgens K. A metatranscriptomics-based assessment of small-scale mixing of sulfidic and oxic waters on redoxcline prokaryotic communities. Environ Microbiol 2019; 21: 584–602.

Bergen B, Naumann M, Herlemann DPR, Gräwe U, Labrenz M, Jürgens K. Impact of a Major inflow event on the composition and distribution of bacterioplankton communities in the Baltic Sea. Front Mar Sci 2018; 5: 1–14.

Bolger A, Lohse M, Usadel B. Trimmomatic: a flexible trimmer for Illumina sequence data. Bioinformatics 2014.

Canfield DE, Stewart FJ, Thamdrup B, De Brabandere L, Dalsgaard T, Delong EF, et al. A cryptic sulfur cycle in oxygen-minimum-zone waters off the Chilean coast. Science (80-) 2010; 330: 1375–8.

Chaumeil P-A, Mussig AJ, Hugenholtz P, Parks DH. GTDB-Tk: a toolkit to classify genomes with the Genome Taxonomy Database. Bioinformatics 2019; 36: 1925–1927.

Elken, J.; Matthäus, W. Physical System Description. In Assessment of climate change for the Baltic Sea basin; 2008.

Feike J, Jürgens K, Hollibaugh JT, Krüger S, Jost G, Labrenz M. Measuring unbiased metatranscriptomics in suboxic waters of the central Baltic Sea using a new in situ fixation system. ISME J 2011; 6: 461–470.

Finn RD, Mistry J, Tate J, Coggill P, Heger A, Pollington JE, et al. The Pfam protein families database. Nucleic Acids Res 2010; 38: D211–D222.

Finn RD, Clements J, Eddy SR. HMMER web server: interactive sequence similarity searching. Nucleic Acids Res 2011; 39: W29–W37.

Gionfriddo C, Capo E, Peterson B, Lin H, Jones D, Bravo A, et al. Hg-cycling Microorganisms in Aquatic and Terrestrial Ecosystems Database v1.01142021. https://doi.org/1025573/serc13105370.v1 2021.

Grote J, Jost G, Labrenz M, Herndl GJ, Jürgens K. Epsilonproteobacteria represent the major portion of chemoautotrophic bacteria in sulfidic waters of pelagic redoxclines of the baltic and black seas. Appl Environ Microbiol 2008; 74: 7546–7551.

Hyatt D, Chen G-L, LoCascio PF, Land ML, Larimer FW, Hauser LJ. Prodigal: prokaryotic gene recognition and translation initiation site identification. BMC Bioinformatics 2010; 11: 119.

Kang DD, Li F, Kirton E, Thomas A, Egan R, An H, et al. MetaBAT 2: an adaptive binning algorithm for robust and efficient genome reconstruction from metagenome assemblies. PeerJ 2019; 7: e7359.

Karlsson M & Lindgren J (2012) www.winsgw.se.

Labrenz M, Sintes E, Toetzke F, Zumsteg A, Herndl GJ, Seidler M, et al. Relevance of a crenarchaeotal subcluster related to Candidatus Nitrosopumilus maritimus to ammonia oxidation in the suboxic zone of the central Baltic Sea. ISME J 2010; 4: 1496–1508.

Langmead B, Salzberg SL. Fast gapped-read alignment with Bowtie 2. Nat Methods 2012; 9: 357–359.

Li H, Handsaker B, Wysoker A, Fennell T, Ruan J, Homer N, et al. The Sequence Alignment/Map format and SAMtools. Bioinformatics 2009; 25: 2078–2079.

Li D, Liu C-M, Luo R, Sadakane K, Lam T-W. MEGAHIT: an ultra-fast single-node solution for large and complex metagenomics assembly via succinct de Bruijn graph. Bioinformatics 2015; 31: 1674–1676.

Liao Y, Smyth GK, Shi W. featureCounts: an efficient general purpose program for assigning sequence reads to genomic features. Bioinformatics 2014; 30: 923–930.

Liem-Nguyen V, Skyllberg U, Björn E. Thermodynamic Modeling of the Solubility and Chemical Speciation of Mercury and Methylmercury Driven by Organic Thiols and Micromolar Sulfide Concentrations in Boreal Wetland Soils. Environ Sci Technol 2017; 51: 3678–3686.

Matsen FA, Kodner RB, Armbrust EV. pplacer: linear time maximum-likelihood and Bayesian phylogenetic placement of sequences onto a fixed reference tree. BMC Bioinformatics 2010; 11: 538.

Muyzer G, Stams AJM. The ecology and biotechnology of sulphate-reducing bacteria. Nat Rev Microbiol 2008; 6: 441–454.

Parks DH, Imelfort M, Skennerton CT, Hugenholtz P, Tyson GW. CheckM: assessing the quality of microbial genomes recovered from isolates, single cells, and metagenomes. Genome Res 2015; 25: 1043–55. Seemann T. Prokka: rapid prokaryotic genome annotation. Bioinformatics 2014.

Selengut JD, Haft DH, Davidsen T, Ganapathy A, Gwinn-Giglio M, Nelson WC, et al. TIGRFAMs and Genome Properties: tools for the assignment of molecular function and biological process in prokaryotic genomes. Nucleic Acids Res 2007; 35: D260–D264.

Skyllberg U, Bloom PR, Qian J, Chung-Min Lin, Bleam§ WF. Complexation of Mercury(II) in Soil Organic Matter: EXAFS Evidence for Linear Two-Coordination with Reduced Sulfur Groups. Environ Sci Technol 2006; 40: 4174–4180.

Soerensen AL, Schartup AT, Skrobonja A, Bouchet S, Amouroux D, Liem-Nguyen V, et al. Deciphering the Role of Water Column Redoxclines on Methylmercury Cycling Using Speciation Modeling and Observations From the Baltic Sea. Global Biogeochem Cycles 2018; 32: 1498–1513.

Stal L, Albertano P, Bergman B, Bröckel K von, Gallon J, Hayes P, et al. BASIC: Baltic Sea cyanobacteria. An investigation of the structure and dynamics of water blooms of cyanobacteria in the Baltic Sea—responses to a changing. Cont Shelf Res 2003; 23: 1695–1714.

Smith SD, Bridou R, Johs A, Parks JM, Elias DA, Hurt RA, et al. Site-directed mutagenesis of HgcA and HgcB reveals amino acid residues important for mercury methylation. Appl Environ Microbiol 2015; 81: 3205–3217

van Vliet DM, von Meijenfeldt FAB, Dutilh BE, Villanueva L, Sinninghe Damsté JS, Stams AJM, et al. The bacterial sulfur cycle in expanding dysoxic and euxinic marine waters. Environ Microbiol 2020.

Wood DE, Lu J, Langmead B. Improved metagenomic analysis with Kraken 2. Genome Biol 2019; 20: 257.
